# Probiotics and Antibiotic-Induced Microbial Aberrations in Children: A Secondary Analysis of a Randomized Clinical Trial

**DOI:** 10.1001/jamanetworkopen.2024.18129

**Published:** 2024-07-05

**Authors:** Thomas H. Dierikx, Anna M. Malinowska, Jan Łukasik, Isolde Besseling-van der Vaart, Clara Belzer, Hania Szajewska, Tim G. J. de Meij

**Affiliations:** 1 Department of Medical Microbiology, Maastricht University Medical Center, Maastricht, the Netherlands; 2 Department of Paediatric Gastroenterology, Amsterdam University Medical Center, University of Amsterdam, Amsterdam, the Netherlands; 3 Laboratory of Microbiology, Wageningen University & Research, Wageningen, the Netherlands; 4 Department of Pediatrics, Medical University of Warsaw, Warsaw, Poland; 5 Winclove Probiotics BV, Amsterdam, the Netherlands; 6 Department of Pediatric Gastroenterology, Emma Children’s Hospital, Amsterdam, the Netherlands

## Abstract

**Importance:**

Probiotics are often considered in children to prevent antibiotic-associated diarrhea. However, the underlying mechanistic effects and impact of probiotics on antibiotic-induced microbiota changes are not well understood.

**Objective:**

To investigate the effects of a multispecies probiotic on the gut microbiota composition in children receiving antibiotics.

**Design, Setting, and Participants:**

This is a secondary analysis of a randomized, quadruple-blind, placebo-controlled clinical trial from February 1, 2018, to May 31, 2021, including 350 children receiving broad-spectrum antibiotics in the inpatient and outpatient settings. Patients were followed up until 1 month after the intervention period. Fecal samples and data were analyzed between September 1, 2022, and February 28, 2023. Eligibility criteria included 3 months to 18 years of age and recruitment within 24 hours following initiation of broad-spectrum systemic antibiotics. In total, 646 eligible patients were approached and 350 participated in the trial.

**Intervention:**

Participants were randomly assigned to receive daily placebo or a multispecies probiotic formulation consisting of 8 strains from 5 different genera during antibiotic treatment and for 7 days afterward.

**Main Outcomes and Measures:**

Fecal stool samples were collected at 4 predefined times: (1) inclusion, (2) last day of antibiotic use, (3) last day of the study intervention, and (4) 1 month after intervention. Microbiota analysis was performed by 16S ribosomal RNA gene sequencing.

**Results:**

A total of 350 children were randomized and collected stool samples from 88 were eligible for the microbiota analysis (54 boys and 34 girls; mean [SD] age, 47.09 [55.64] months). Alpha diversity did not significantly differ between groups at the first 3 times. Shannon diversity (mean [SD], 3.56 [0.75] vs 3.09 [1.00]; *P* = .02) and inverse Simpson diversity (mean [SD], 3.75 [95% CI, 1.66-5.82] vs −1.31 [95% CI, −3.17 to 0.53]; *P* = 1 × 10^−4^) indices were higher in the placebo group compared with the probiotic group 1 month after intervention. Beta diversity was not significantly different at any of the times. Three of 5 supplemented genera had higher relative abundance during probiotic supplementation, but this difference had disappeared after 1 month.

**Conclusions and Relevance:**

The studied probiotic mixture had minor and transient effects on the microbiota composition during and after antibiotic treatment. Further research is needed to understand their working mechanisms in manipulating the microbiome and preventing antibiotic-associated dysbiosis and adverse effects such as antibiotic-associated diarrhea.

**Trial Registration:**

ClinicalTrials.gov Identifier: NCT03334604

## Key Points

**Question:**

What is the effect of a multispecies probiotic on antibiotic-induced gut microbiota aberrations in children?

**Findings:**

In this randomized clinical trial of 88 children, the studied probiotic mixture had minor and transient effects on the microbiota composition during and after antibiotic treatment. Three of 5 supplemented genera had higher relative abundance during probiotic supplementation, which subsequently reverted to baseline levels at 1-month follow-up.

**Meaning:**

Use of the studied probiotic formulation may prevent antibiotic-induced adverse effects via gut microbiota modulation, but further research combining multiomics technology is warranted.

## Teaser

This randomized clinical trial assesses the effect of a multispecies probiotic supplement on antibiotic-associated diarrhea among patients aged 18 years and younger who are receiving antibiotics.

## Introduction

Antibiotics are among the most frequently prescribed drugs in children.^1,2^ Currently, antibiotic prescription rates range from 0.5 to 1.6 courses per child-year in Western countries.^3^ Exposure to antibiotics results in a decreased diversity and abundance of commensal microorganisms with a concurrent increase of pathogens in the gut microbiota.^4,5^ In early life, gut microbiota play an important role in multiple physiologic processes, including priming and development of the immune system and digestion.^6^ Consequently, antibiotic-induced dysbiosis, particularly during early childhood, has been associated with long-term health outcomes such as obesity, asthma, Crohn disease, and type 1 diabetes.^7,8,9,10,11^ In the short term, the most common adverse effect is antibiotic-associated diarrhea (AAD).^4^ Since prescription of antibiotics cannot always be avoided, it is pivotal to study interventions that could prevent, mitigate, or quickly restore antibiotic-induced microbial alterations and adverse events in children.

The most thoroughly studied intervention to prevent harms of antibiotic use consists of probiotics, defined as “live microorganisms that, when administered in adequate amounts, confer a health benefit on the host.”^12^ Recently, we demonstrated in a randomized clinical trial (RCT) that supplementation of a multispecies probiotic in antibiotic-exposed children resulted in a significantly decreased risk of diarrhea.^13^ It has been hypothesized that concomitant supplementation of probiotics during antibiotic therapy may protect against such antibiotic-induced adverse events by modulating the microbiota.^14,15^ However, the presumed underlying protective mechanisms of probiotics, including their mitigating effects on antibiotic-induced microbiota aberrations, have not yet been thoroughly studied in children.^14^ Therefore, we longitudinally assessed the effect of a multispecies probiotic on the microbiota composition in children receiving antibiotics.

## Methods

This secondary analysis of an RCT followed the Consolidated Standards of Reporting Trials (CONSORT) reporting guideline. The study was approved by the Bioethics Committees of the Medical University of Warsaw, Warsaw, Poland, and Amsterdam University Medical Center, Amsterdam, the Netherlands. Written informed consent was obtained from all parents for children aged 0 to 11 years, children and parents for children aged 12 to 15 years, and children only for those 16 years or older.

### Study Design

We conducted a quadruple-blind, placebo-controlled RCT in 3 Dutch and 2 Polish hospitals.^16^ The primary aim of the trial was to assess the effect of a multispecies probiotic on the incidence of AAD, for which results were reported previously.^13^ This RCT compared a placebo group with a probiotic supplement group. We obtained fecal samples from children in the RCT to longitudinally describe the effects of a multispecies probiotic on the gastrointestinal tract microbiota composition in children receiving antibiotics. The trial protocol is provided in Supplement 1.

### Participants

All children and adolescents aged 3 months to 18 years (hereinafter referred to as children) starting broad-spectrum oral or intravenous antibiotic treatment were eligible for participation. Children were recruited from February 1, 2018, to May 31, 2021. Children were eligible if recruited within 24 hours following initiation of antibiotics. Children were only included in the microbiota analysis if the child or parents collected 2 or more fecal samples and if children were adherent with the study protocol. Children were considered adherent if they received over 75% of the recommended doses of the study product. Exclusion criteria have been described previously.^13^

### Procedures and Interventions

Children received either a multispecies probiotic or placebo twice a day for the duration of antibiotic treatment and the 7 subsequent days, up to a maximum of 17 days, starting within 24 hours of the first antibiotic dose. Randomization and masking procedures have been described previously.^13^ The multispecies probiotic (Ecologic AAD 612; Winclove Probiotics BV) contained 8 bacterial strains: *Bifidobacterium bifidum* W23, *Bifidobacterium lactis* W51, *Lactobacillus acidophilus* W37, *L acidophilus* W55, *Lacticaseibacillus paracasei* W20, *Lactiplantibacillus plantarum* W62, *Lacticaseibacillus rhamnosus* W71, and *Ligilactobacillus salivarius* W24, for a total dose of 5 billion colony-forming units (CFU) per sachet (10 billion CFU/d). Dutch participants collected fecal samples in 10-mL sterile containers (Stuhlgefäß; Greiner) that were immediately frozen after collection. Samples collected at home were picked up at home by one of the researchers (T.H.D.) and transported to the hospital, where the samples were stored at −20 °C. Polish participants collected stool samples in a commercially available kit container (OMNIgene•GUT; Omnitek) containing a DNA stabilization buffer and were sent to the hospital, where samples were immediately stored at −80 °C. Fecal samples were collected at 4 times: (1) first stool following inclusion, (2) the last day of antibiotic treatment, (3) the last day of the placebo or probiotic supplementation, and (4) 1 month after termination of placebo or probiotic supplementation.

### Sample Handling

Samples were analyzed in the Laboratory of Wageningen University & Research, Wageningen, the Netherlands, using procedures described previously.^17^ A total of 250 μg of each fecal sample was homogenized using bead beating, and DNA was extracted with a commercially available system (Maxwell 16; Promega Corporation) according to the manufacturer’s protocol. Polymerase chain reaction was performed to amplify the V4 hypervariable regions of the bacterial 16S ribosomal RNA (rRNA) gene using barcoded primers 515F (5′-GTGCCAGCMGCCGCGGTAA-) and 806R (5′-GTGCCAGCMGCCGCGGTAA-). Six libraries were constructed by pooling 70 uniquely barcoded samples per library. Quality control was assessed by adding negative controls and artificial mock communities to libraries. Amplicon mixture was sequenced using a commercially available platform (HiSeq 2000; Illumina, Inc). Data processing used a semantic framework (NG-Tax [open source]) with default settings, apart from read length, which was set to 100 base pairs.^18^ Taxonomic assignment of amplicon sequence variants (ASVs) was performed using a reference database (SILVA_138.1).^19,20^ All laboratory analyses were performed by researchers unaware of participants’ allocation to the probiotic intervention or control group.

### Outcomes

The primary outcome of the original trial was the incidence of AAD, the results of which have been published previously.^13^ The effects of probiotics on the gastrointestinal microbiota composition in children receiving antibiotics was a secondary outcome of this trial, the data for which are presented in the present study. The objective was to analyze differences in changes of microbiota composition between the placebo and probiotic group over time. This was followed by cross-sectional comparison between the 2 groups at all time points separately.

### Microbiome Data Analysis

All analyses were performed in R software, version 4.2.1 (R Project for Statistical Computing) using the microbiome, phyloseq,^21^ and vegan^22^ packages. All samples with read counts lower than those for the negative controls were excluded from further analysis. Taxa not assigned to any phylum were removed from the dataset. To ensure that the contaminant and rare taxa were removed, only taxa with abundance over 0.25% in at least 1 sample were left in the dataset.^23^ The median number of reads per sample for the 16S rRNA gene amplicon dataset was 175 933 (range, 2273-2 106 395). In total, 1471 different ASVs and 180 genera were identified.

To quantify the breadth of individual microbiome diversity, we calculated alpha diversity (Shannon and inverse Simpson) indices for each sample on ASV level prior to filtering out rare taxa. To analyze microbiota community changes over time, beta diversity was assessed separately for each of the study groups using the principal coordinates analysis method with Bray-Curtis distance on ASV taxonomic level. Moreover, to analyze differences in microbial beta diversity between groups, the analyses were performed separately for each collection time. All analyses of gut microbiota composition were performed based on the relative abundances of the taxa.

### Statistical Analysis

Data were analyzed between September 1, 2022, and February 28, 2023. Descriptive statistics were used to present the participants’ baseline characteristics of the 2 groups. For dichotomous data, the χ^2^ test was used. For continuous data, the unpaired 2-tailed *t* test and Mann-Whitney test were used for normally and nonnormally distributed data, respectively. All statistical tests were 2 tailed and were performed with a 5% level of significance; *P* < .05 was considered statistically significant. First, changes in diversity and abundance over time were compared, followed by cross-sectional comparison at the 4 times separately.

To assess changes of alpha diversity over time in antibiotic-exposed children with or without probiotic supplementation, linear mixed models adjusted for age and country were used (lme4 package in R, version 4.2.1). To account for repeated measurements, participants’ identifications were used as random effects. Evaluation of the statistical significance of the time term in the model was assessed by comparing (using χ^2^ statistics) the built model with the model where time was dropped. In case of significant results, a post hoc Tukey test was used (emmeans package in R, version 4.2.1). All *P* values were corrected using the false discovery rate approach, and *P* < .05 was considered statistically significant. Next, statistical significance of differences in the compositional change trajectories between groups was assessed by testing the interaction effect between time point and group coding using linear mixed models with participants’ identifications as random effects. The analysis was adjusted for age and country. Permutational analysis of variance (PERMANOVA) was used to test whether the bacterial composition was related to study group and time and whether there was an interaction between time and study group. Then, to assess which times differed significantly from one another, linear mixed-effects models were used in which the distances between points on the coordinate axes were compared between times within each group. Moreover, overall changes in beta diversity were studied by calculating dissimilarity indices. This was done by comparing the Bray-Curtis distance between samples of the same participants collected in times 1 and 2, 1 and 3, and 1 and 4. Dissimilarity indices were then compared between the groups, and linear mixed-model effects were used to check whether these changes are differentiated by group and time change interaction.

To compare the differences in microbiota composition changes between study groups, fold changes in each group between time 1 and times 2, 3, and 4 were calculated by dividing the relative abundance of the taxa in a later time by the relative abundance in an earlier time. Since microbiome data are zero inflated, a zero replacement strategy was applied prior to fold changes calculation. All zeros were replaced by a constant value that was equal to 65% of the detection limit.^24^ A binary logarithm was then calculated for fold changes, and these values were compared between groups within each time change (from time 1 to 2, 1 to 3, and 1 to 4). The plots were prepared using the ggplot2 and microViz packages (R, version 4.2.1).

## Results

**Figure 1.**
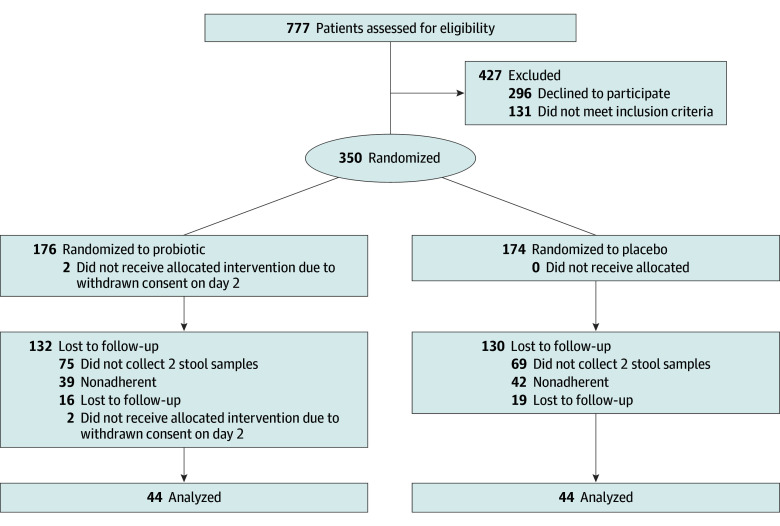
Study Flowchart

Among the 350 children included in the original RCT, 88 (44 in the probiotic group and 44 in the placebo group; 54 boys and 34 girls; mean [SD] age, 47.09 [55.64] months) were adherent to the study protocol regarding collection of at least 2 stool samples with enough reads between February 1, 2018, and May 31, 2021 (Figure 1). A total of 19 samples had to be excluded from the analyses because of low read counts. Participants’ characteristics were comparable between the 2 groups (Table). Characteristics of participants included in this study and participants who dropped out after inclusion in the original trial are provided in eTable 1 in Supplement 2. Race and ethnicity data were not collected. A limited number of children in each group (13 in the placebo group [29.5%] and 7 in the probiotic group [15.9%]) had diarrhea, with 10 in the placebo group (22.7%) and 6 in the probiotic group (13.6%) with AAD.

**Table.** Participant Characteristics^a^

Characteristic	Patient group	
	Placebo (n = 44)	Probiotic (n = 44)
Age, median (IQR), mo	18.04 (25.78)	27.14 (90.88)
Sex		
Female	18 (40.9)	16 (36.4)
Male	26 (59.1)	28 (63.6)
Participants included in the Netherlands	35 (79.5)	37 (84.1)
Inpatient	24 (54.5)	23 (52.3)
Length of hospital stay, median (IQR), d	2.00 (3.25)	1.50 (3.00)
Antibiotic treatment duration, median (IQR), d	7.0 (3.0)	7.5 (3.0)
Antibiotic administration route		
Only oral	32 (72.7)	31 (70.5)
Only intravenous	1 (2.3)	4 (9.1)
Intravenous followed by oral	11 (25.0)	9 (20.5)
Reason for treatment		
URTI	11 (25.0)	2 (4.5)
LRTI	11 (25.0)	14 (31.8)
UTI	14 (31.8)	8 (18.2)
Joint	1 (2.3)	0
Lymphadenitis	1 (2.3)	3 (6.8)
GI tract	0	2 (4.5)
Skin	4 (9.1)	9 (20.5)
Nervous system	0	2 (4.5)
Other	2 (4.5)	4 (9.1)
Antibiotic^b^		
Second-generation cephalosporin	4 (9.1)	1 (2.3)
Third-generation cephalosporin	2 (4.5)	5 (11.4)
Aminopenicillin	13 (29.5)	16 (36.4)
Amoxicillin with clavulanic acid	28 (63.6)	24 (54.6)
Clindamycin	0	1 (2.3)
Other	2 (4.6)	3 (6.8)
Two concomitant antibiotics	1 (2.3)	3 (6.8)
Change of antibiotic class	4 (9.1)	6 (13.6)
Diarrhea cases^c^	13 (29.5)	7 (15.9)
AAD cases^d^	10 (22.7)	6 (13.6)

Abbreviations: AAD, antibiotic-associated diarrhea; GI, gastrointestinal; LRTI, lower respiratory tract infection; URTI, upper respiratory tract infection; UTI, urinary tract infection.

^a^ Unless otherwise indicated, data are expressed as No. (%) of patients.

^b^ Percentages do not add to 100 because some participants were given a combination of antibiotics. Possible combinations include second- and third-generation cephalosporin (n = 1), third-generation cephalosporin and amoxicillin with clavulanic acid (n = 3), third-generation cephalosporin and aminopenicillin (n = 1), aminopenicillin and amoxicillin with clavulanic acid (n = 1), aminopenicillin and other antibiotic (n = 1), amoxicillin with clavulanic acid and clindamycin (n = 1), and amoxicillin with clavulanic acid and other antibiotic (n = 3).

^c^ Diarrhea was defined as 3 or more loose or watery stools in a 24-hour period regardless of the etiology.

^d^ AAD was defined as 3 or more loose or watery stools in a 24-hour period, caused either by *Clostridium difficile* or of otherwise unexplained etiology, after testing for common, predefined diarrheal pathogens.

### Differences in Microbial Diversity Between the Placebo and Probiotic Groups

**Figure 2.**
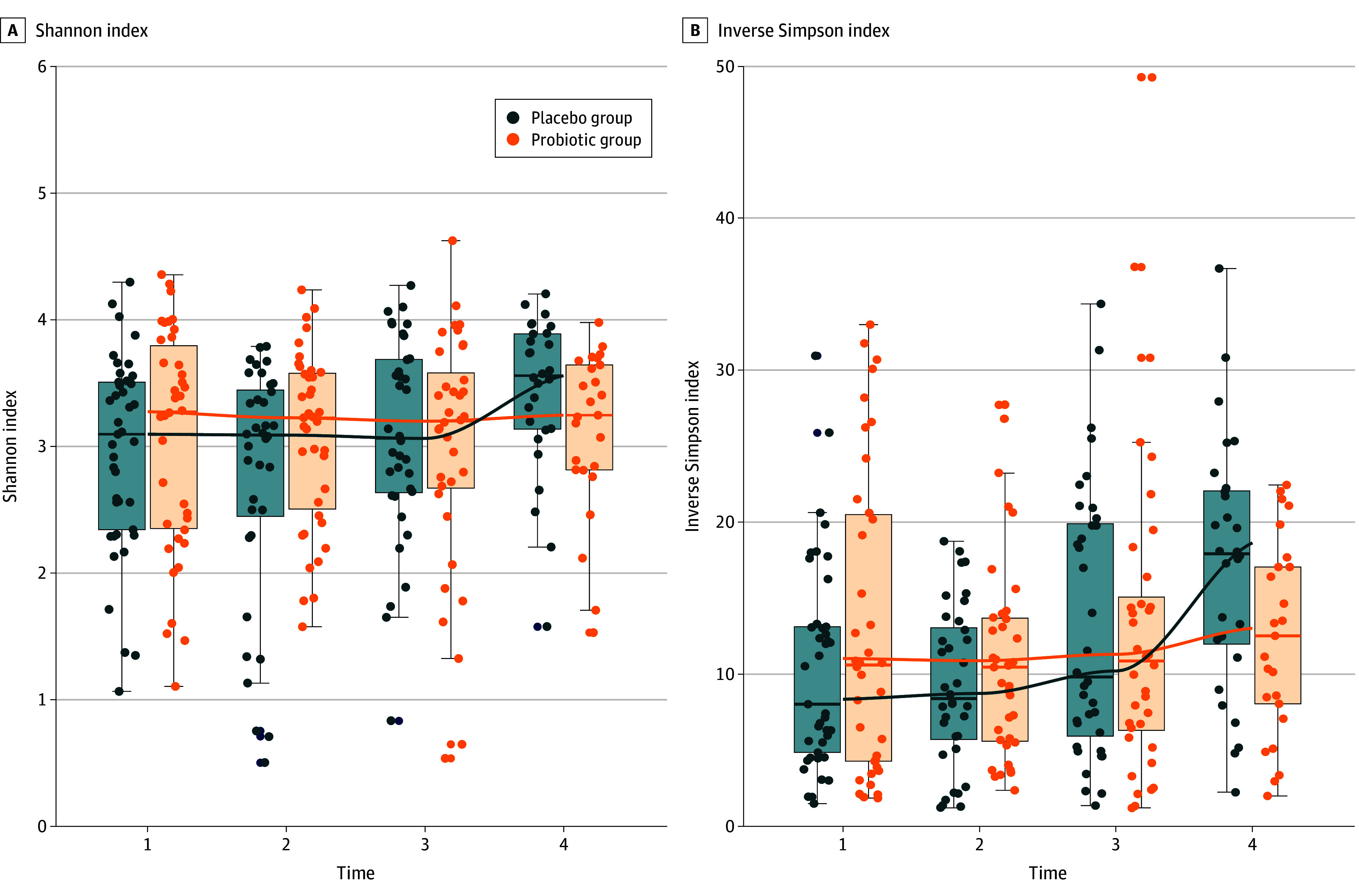
Alpha Diversity Trajectory of alpha diversity changes in time differed between groups since the interaction term between group and time was statistically significant in linear mixed models explaining the Shannon index and the inverse Simpson index. A, Mean (SD) Shannon indices for the placebo compared with probiotic groups. B, Mean (SD) inverse Simpson indices for the placebo compared with probiotic groups. Both diversity indices were higher at time 4 (1-month follow-up) compared with time 1 (first sample after inclusion) and time 2 (last day of antibiotic treatment) in the placebo group. Boxes indicate upper and lower quartiles; horizontal lines in boxes, median; and whiskers, minimum and maximum.

No differences were found in change of any of the alpha diversity indices during the first 2 collection times in both study groups. In the placebo group, higher values were found at time 4 compared with time 2 in the Shannon diversity index (mean [SD], 3.56 [0.75] vs 3.09 [1.00]; *P* = .02) and the inverse Simpson index (mean [SD], 3.75 [95% CI, 1.66-5.82] vs −1.31 [95% CI, −3.17 to 0.53]; *P* < .001) (Figure 2). Such changes across times were not noticed in the probiotic group, and regression analysis showed that the study groups differed in the trajectories of changes in both the Shannon index (β coefficients, −0.22 [95% CI, −0.56 to 0.12] at time 1; −0.39 [95% CI, −0.74 to −0.04] at time 2; −0.09 [95% CI, −0.44 to 0.26] at time 3; and 0.14 [95% CI, −0.23 to 0.51] at time 4; *P* = .05 for interaction) and inverse Simpson index (β coefficients, −2.57 [95% CI, −5.94 to 0.81] for time 1; −3.55 [95% CI, −6.99 to −0.09] for time 2; 0.28 [95% CI, −3.17 to 3.75] for time 3; and 3.72 [95% CI, 0.10-7.34] for time 4; *P* < .001 for interaction). Cross-sectional comparison between the placebo and probiotic groups revealed no differences in Shannon diversity and inverse Simpson indices in the first 3 times. The Shannon diversity index was higher in the placebo group compared with the probiotic group at time 4 (mean [SD], 3.56 [0.75] vs 3.25 [0.83]; *P* = .048) (Figure 2A), as was the inverse Simpson index (17.92 [10.08] vs 12.52 [9.00]; *P* = .03) (Figure 2B).

**Figure 3.**
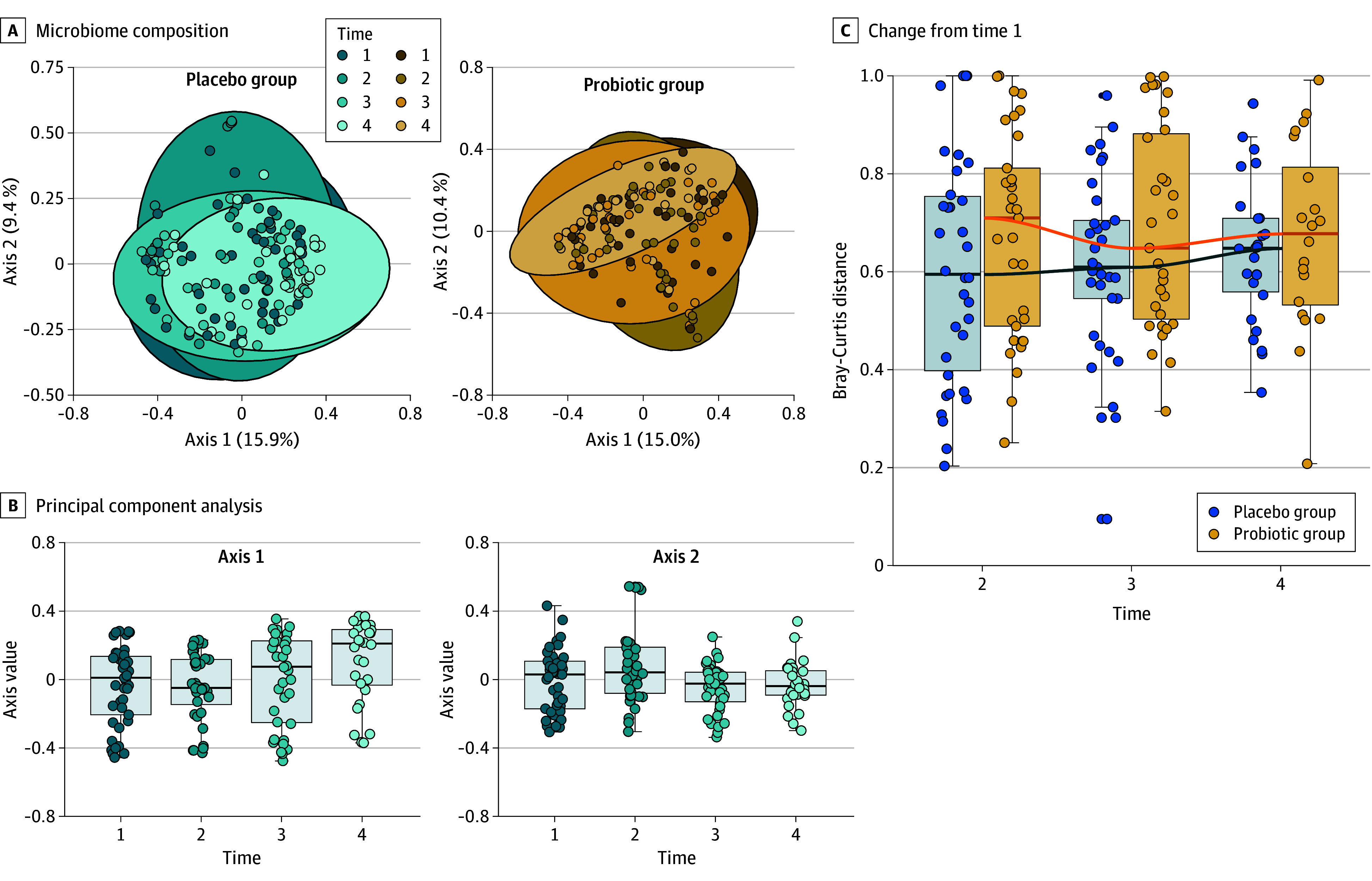
Beta Diversity A, Overall microbiome composition was associated with time in the placebo group (*R*^2^ = 1.76%; *P* = .004) but not in the probiotic group (*P* = .08). B, Samples from time 4 were significantly further from samples in times 1 (*P* = .001), 2 (*P* < .001), and 3 (*P* = .02) on the first axis in the placebo group. Samples from time 2 were significantly further from samples in times 1 (*P* = .04), 3 (*P* = .005), and 4 (*P* = .04) on the second axis in the placebo group. C, The dispersion of samples from each time point in the probiotic group was not equal, therefore the association of overall microbiome composition with time could not be assessed in this group (time effect *P* > .99 for the probiotic group and *P* = .70 for the placebo group; *P* = .53 for interaction in the probiotic group). Boxes indicate upper and lower quartiles; horizontal lines in boxes, median; and whiskers, minimum and maximum.

Beta diversity analysis showed that time was associated with overall microbiome composition in the placebo group (*R*^2^ = 1.76%; *P* = .004) (Figure 3A). In the multidimensional space, samples from time 4 were significantly further from samples in times 3 (β coefficient, 0.13 [95% CI, 0.07-0.19]; *P* = .02), 2 (β coefficient, 0.03 [95% CI, −0.03 to 0.08]; *P* < .001), and 1 (β coefficient, −0.02 [95% CI, −0.07 to 0.04]; *P* = .001) (Figure 3B) on the first axis. The dispersion of samples from each time in the probiotic group was not equal (mean difference in distance to centroid between times 2 and 4, −0.05 [95% CI, −0.09 to −0.01; *P* = .008]; between times 3 and 4, −0.04 [95% CI, −0.08 to 0.00; *P* = .05] for homogeneity of variance test); therefore, the association of overall microbiome composition with time could not be assessed in this group. However, PERMANOVA analysis indicated there was no interaction effect between groups and time (F = 0.56; *P* = .97), indicating there were no significant differences between groups in beta diversity changes over time.

Dissimilarity indices also showed that microbiota composition in time 4 was dissimilar compared with the microbiota composition in time 1 in both groups. The composition in times 2 and 3 were equally dissimilar from time 1 as well (Figure 3C). Cross-sectional analysis of the beta diversity showed no difference between the placebo and probiotic groups at all 4 times (eFigure 1 in Supplement 2).

### Differences in the Changes of Taxonomic Composition Between the Placebo and Probiotic Groups

**Figure 4.**
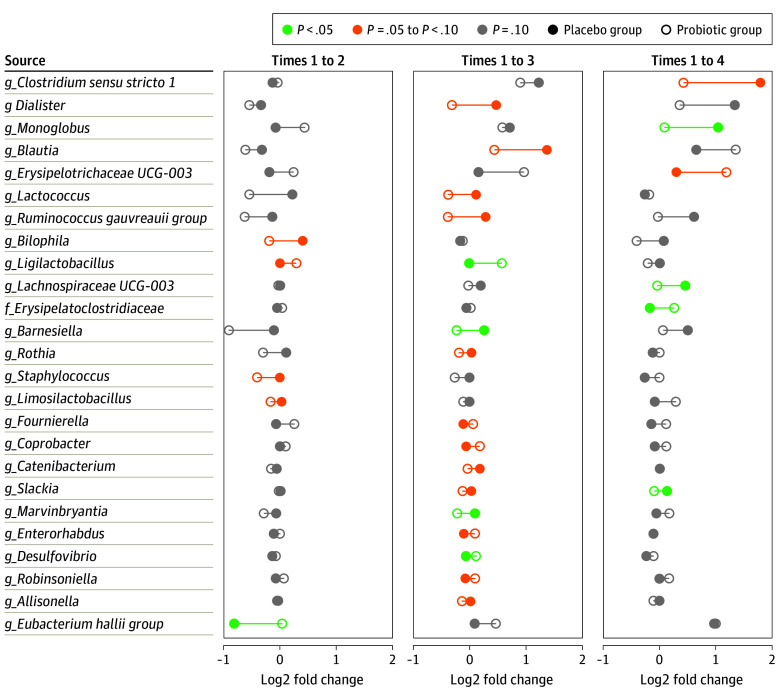
Log Fold Changes Compared With Time 1 Differences between the placebo and probiotic groups in changes in relative abundance of taxa between times with *P* < .10. A point below 0 indicates a decrease compared with time 1, whereas a point above 0 indicates an increase compared with time 1. Lower case f indicates family; lower case g, genus.

Compared with time 1, there was a decrease at time 2 in the abundance of the genus *Eubacterium* in the placebo group (mean drop of 0.03% [95% CI, −0.10% to 0.04%]), whereas no changes were observed in the probiotic group (0 [95% CI, 0-0]; *P* = .05 for the comparison of log fold changes between groups). There were no significant differences between the study groups in changes of other genera in time 2 compared with time 1. At time 3, there were significant differences between the placebo and probiotic groups in the change of relative abundance of 4 genera compared with time 1. The increase in *Ligilactobacillus* species was significantly greater in the probiotic group (0.16% [95% CI, −0.05% to 0.37%]) compared with the placebo group (0 [95% CI, 0-0]; *P* = .02). Furthermore, changes in *Desulfovibrio* species (−0.02% [95% CI, −0.05% to 0.01%] in the placebo group vs −0.01% [95% CI, −0.01% to 0.03%] in the probiotic group; *P* = .049), *Barnesiella* species (0.18% [95% CI, −0.20% to 0.56%] in the placebo group vs −0.22% [95% CI, −0.65% to 0.22%] in the probiotic group; *P* = .02), and *Marvinbryantia* species (0.01% [95% CI, −0.0004% to 0.02%] in the placebo group vs −0.03% [95% CI, −0.08% to 0.01%] in the probiotic group; *P* = .03) were significantly different between groups. At time 4, the change in relative abundance between the placebo and probiotic groups was significantly different for *Monoglobus* species (0.18% [95% CI, 0.06%-0.29%] in the placebo group vs 0.01% [95% CI, −0.05% to 0.07%] in the probiotic group; *P* = .005), *Lachnospiraceae UCG-003* species (0.09% [95% CI, −0.02% to 0.19%] in the placebo group vs −0.01% [95% CI, −0.02% to 0.01%] in the probiotic group; *P* = .04), and *Slackia* species (0.02% [95% CI, −0.01% to 0.04%] in the placebo group vs −0.01% [95% CI, −0.03% to 0.01%] in the probiotic group; *P* = .049). These reported *P* values along with the reported increase or decrease refer to the comparison between differences in log fold changes between the placebo and probiotic groups as shown in Figure 4.

### Cross-Sectional Differences in Taxonomic Composition Between the Placebo and Probiotic Groups

Regarding the taxonomic composition of the microbiota at the phylum level, *Verrucomicrobiota* had a higher relative abundance in the placebo group compared with the probiotic group at time 4 (mean [SD], 0.23 [0.06] vs 0.01 [0.02]; *P* = .04) (eFigure 2 in Supplement 2). At the genus level, significant differences were found between the 2 groups in the abundance of 18 different taxa spread across the 4 times (eFigure 3 in Supplement 2). At time 1, the abundance of 5 genera was significantly different between the 2 groups. Regarding the genera present in the supplemented probiotic formulation, a higher abundance was found in *Ligilactobacillus* at time 2 (mean [SD], 0 [0.01] vs 0.002 [0.01]; *P* = .008) and time 3 (mean [SD], 0 [0] vs 0.001 [0.003]; *P* = .006) in the probiotic group compared with the placebo group. Also, a higher abundance was found in the genus *Lactiplantibacillus* (mean [SD], 0 [0] vs 0.001 [0.003]; *P* = .006) and *Lactobacillus* (mean [SD], 0 [0.002] vs 0.004 [0.01]; *P* = .004) at time 3. No differences in *Bifidobacteria* were found between the 2 groups at any of the 4 times. No significant differences were found in the supplemented genera at time 4, which corresponds to 1 month after cessation of intake of the study material. An overview of all observed genera included in the placebo and probiotic groups is given in eTables 2 to 4 in Supplement 3 along with adjusted *P* values.

## Discussion

In this secondary analysis of an RCT, we investigated the effect of probiotic supplementation on antibiotic-associated microbiota aberrations in children. Alpha diversity did not differ between the 2 groups during the intervention period, but the Shannon diversity and inverse Simpson indices were higher in the placebo group 1 month after cessation of the intervention. The studied probiotics had minor and transient effects on the microbiota, including increased abundance of 3 of 5 genera during supplementation.

It is hypothesized that probiotics mitigate antibiotic-induced gut microbiota aberrations and consequently decrease antibiotic-related adverse effects such as AAD. However, mechanistic evidence is limited, particularly in children.^14,15^ In this study, we did not observe major effects of antibiotics on diversity indices in either of the groups, in contrast to what was expected and to previous studies in children.^4,25^ This may be due to most of the baseline stool samples being collected after ingestion of 1 or more dose of antibiotics, since it was not feasible or ethical to postpone start of antibiotic therapy until after the first stool sampling. The first antibiotic doses may consequently have already affected the microbiota composition measured in the baseline sample, as supported by the differences in 5 genera between groups at time 1. One may therefore speculate that the alpha diversity at baseline, before starting antibiotic therapy, was in reality higher than measured in our baseline samples. If that indeed was the case, the alpha diversity would first decrease during antibiotic treatment (time 2). Then, in the placebo group, the alpha diversity would increase or return to baseline at time 4, and not in the probiotic group. A 2018 study^26^ concluded that probiotic supplementation led to slower reconstitution and lower alpha diversity up to 5 months compared with spontaneous recovery, although that study did include a very limited number of participants. A recent meta-analysis^28^ also concluded that probiotic supplementation during antibiotic therapy did not impact diversity indices. However, a more diverse microbiome full of harmful bacteria may be less healthy compared with a less diverse microbiome consisting of healthy bacteria. Which taxa are good or bad bacteria remains an ongoing debate. Therefore, a more diverse microbiome is not necessarily a healthier one, and a lack of overall net change in diversity does not necessarily mean that there are not meaningful changes.

Another placebo-controlled trial in antibiotic-exposed adults (n = 136), supplementing the intervention arm with *L paracasei* CNCM I-1518 and *L rhamnosus* CNCM I-3690 for 28 days, including the 14-day antibiotic treatment,^27^ also showed that probiotic supplementation resulted in increased abundance of the supplemented probiotics, in line with our results. However, the investigators found a reduced degree of antibiotic-induced aberrations and earlier restoration within 28 days after antibiotic cessation,^27^ which was not clearly observed in our study.

Several other studies^14,28^ of the effects of probiotic supplementation on the gut microbiota during antibiotic treatment have shown conflicting results regarding diversity indices, microbiota composition, and recovery time. However, these previous studies included different study populations, including adults or neonates; other types, doses, and durations of probiotics and antibiotics; and stool samples collected at different times and analyzed by different methods.^14^ These differences limit the possibility of reliably comparing results of these studies with our data.

The supplemented genera *Ligilactobacillus*, *Lactiplantibacillus*, and *Lactobacillus* were found in higher abundance among children receiving probiotics. In addition to colonization of the supplemented probiotic strains, administration of probiotics may result in a broad range of changes in the taxonomic composition and function of the microbiome community.^29^ Consequently, probiotics may hypothetically have the potential to prevent antibiotic-associated adverse effects such as diarrhea.^14^ There are several mechanisms hypothesized for the development of diarrhea following administration of antibiotics. Antibiotics may lead to decreased intestinal epithelium function and a leaky gut, with increased risk for diarrhea.^25^ *Lactobacillus* species may prevent antibiotic-induced epithelium dysfunction and stimulate the gut barrier integrity.^14^ Antibiotic exposure leads to microbiota aberrations, accumulation of carbohydrates, and consequently reduced levels of short-chain fatty acids (SCFAs). As SCFAs promote the absorption of water from the colon, a decrease in SCFAs provoke diarrhea.^14^ In children receiving probiotic supplements, we also observed a higher abundance of genus *Coprococcus* (eFigure 3 in Supplement 2). As *Coprococcus* species and the different lactic acid bacteria that metabolize carbohydrates as a main carbon source play an important role in the digestion of carbohydrates into SCFAs, increased abundance of these taxa may lead to increased SCFA concentrations. This will stimulate water absorption and decrease the risk for antibiotic-induced diarrhea.^14^ Increased levels of SCFAs were found after supplementation of different *Lactobacillus* species in adults and animal models.^30,31^ These effects of probiotics on microbiota composition and function may consequently prevent AAD. Due to the limited number of children with diarrhea in this study (13 in the placebo group and 7 in the probiotic group), we were unable to perform subgroup analyses in children with and without diarrhea to further investigate the role of probiotics in AAD.

Studies measuring metabolite levels in antibiotic-exposed children receiving probiotics are lacking. Given the limited evidence, future mechanistic studies focusing on the microbiota function are warranted to elucidate the exact working mechanisms of probiotics. This may elucidate the optimal types, combination, dosing, and duration of probiotic therapy. These studies should also focus on long-term health outcomes of probiotic exposure, as this has not been studied.

### Strengths and Limitations

Strengths of this study include the randomized, placebo-controlled design of the study, which allowed us to compare probiotic-exposed participants with controls, and standardized collection of a relatively large number of samples. Additionally, this study is one of the first, to our knowledge, to focus on the longitudinal effects of a multispecies probiotic in antibiotic-exposed children.

This study also has several limitations. Due to the limited number of children with diarrhea, we were unable to investigate differences in the microbiota between children with diarrhea (nonresponders) and children without diarrhea (responders). Additionally, as reported previously, the baseline sample may have been affected by antibiotics, as it was collected after the first antibiotic dose in most cases. It was impossible and unethical to postpone antibiotic initiation until collection of the first fecal sample. The infection for which antibiotics were prescribed may have affected the gut microbiota composition, especially in those cases with a gastrointestinal tract infection. Furthermore, not all children recruited in the initial trial focusing on AAD incidence were included in this part of the study, as not all participants collected at least 2 stool samples and were adherent to the study protocol. This led to a loss of power to study the microbiota as an outcome. Regardless, this remains the largest study to investigate the effect of probiotics on the microbiota in children receiving antibiotics. The high number of dropouts may have introduced confounding, and although this study was an RCT, residual confounding cannot be completely ruled out. In the present study, most participants were recruited from Dutch centers, whereas most participants in the original trial were recruited in Poland. Differences in antibiotic treatment indication and duration and national guidelines has led to differences in participants who were included in the original trial and participants who dropped out. There was a broad age range of children included in our study, and different types of antibiotics were prescribed for different indications, potentially affecting the results. Other variables impacting the microbiota that were not measured in this study, such as diet, may have biased the results. Only 16S rRNA gene sequencing was performed to study the microbiota composition; metabolomics analysis will be performed on collected samples in the future, allowing insight into microbial function rather than only composition.

## Conclusions

In this secondary analysis of an RCT, the studied probiotics had minor and transient effects on the microbiota compared with placebo, including increased abundance of 3 of 5 supplemented genera during supplementation, which subsequently reverted to baseline levels at 1-month follow-up. Alpha and beta diversity were not different during probiotic supplementation, but both the Shannon diversity and inverse Simpson indices were increased in the placebo group at 1-month follow-up. It therefore remains debated whether probiotics have beneficial effects on antibiotic-induced microbiota composition aberrations. Future studies with adequate baseline samples and homogenous study populations that also focus on the function of the microbiota and the association between the microbiota and clinical outcomes are needed to assess whether observed transient effects on taxonomic composition and effects on diversity have a mechanistic role in protection against antibiotic-induced adverse effects, including AAD.

## Notes

Supplement 1.
Trial Protocol

Supplement 2. **eTable 1.** Characteristics of Included Participants and Participants Lost to Follow-Up **eFigure 1.** Beta Diversity at All 4 Times **eFigure 2.** Relative Abundance at Phylum Level **eFigure 3.** Relative Abundance of Genera With Significantly Different Relative Abundance

Supplement 3. **eTable 2.** Dataset 1 **eTable 3.** Diarrhea Analyses **eTable 4.** AAD

Supplement 4.
Multispecies Probiotic in AAD Study Group

Supplement 5.
Data Sharing Statement
