# Supplementary material for: Probiotics and Antibiotic-Induced Microbial Aberrations in Children: A Secondary Analysis of a Randomized Clinical Trial
Source: JAMA Netw Open. 2024 Jul 5;7(7):e2418129. doi: 10.1001/jamanetworkopen.2024.18129 (PMC11227081; doi:10.1001/jamanetworkopen.2024.18129)
Supplement: Supplement 1. — Trial Protocol [file jamanetwopen-e2418129-s001.pdf]

Research protocol

The Influence of Probiotics on Pediatric Antibiotic-associated Diarrhoea

IPAD study

Version 8
30-11-2020

The Influence of Probiotics on Pediatric Antibiotic-associated Diarrhoea – IPAD study

**Protocol ID**	**NL69225.029.19**
**Short title**	IPAD study
**EudraCT number**	Not applicable
**Version**	8.0
**Date**	30-11-2020
**Project leaders**	Dhr. dr. T.G.J. de Meij<br>Amsterdam UMC, location VUmc, Amsterdam<br>Department of Pediatric Gastroenterology<br>Room: ZH 9D 38<br>Postbus 7057, 1007 MB Amsterdam, The Netherlands<br>t.demeij@vumc.nl<br><br>Dhr. prof. Dr. M.A. Benninga<br>Amsterdam UMC, location VUmc, Amsterdam<br>Department of Pediatric Gastroenterology<br>m.a.benninga@amsterdamumc.nl
**PhD student**	Dhr. T.H. Dierikx<br>Amsterdam UMC, location VUmc, Amsterdam<br>Department of Pediatric Gastroenterology<br>Room: ZH 9D 36<br>Postbus 7057, 1007 MB Amsterdam, The Netherlands<br>t.dierikx@amsterdamumcumc.nl
**Principal investigator**	Dhr. dr. T.G.J. de Meij<br>Amsterdam UMC, location VUmc, Amsterdam<br>Department of Pediatric Gastroenterology<br>Room: ZH 9D 38<br>Postbus 7057, 1007 MB Amsterdam, The Netherlands<br>t.demeij@amsterdamumc.nl
**Participating centres:**	Amsterdam UMC, location VUmc<br>De Boelelaan 1117, 1081 HV, Amsterdam<br><br>Amsterdam UMC, location AMC<br>Meibergdreef 9, 1105 AZ, Amsterdam<br><br>Onze Lieve Vrouwe Gasthuis, locatie Oost<br>Oosterpark 9, 1091 AC, Amsterdam<br><br>Onze Lieve Vrouwe Gasthuis, location West<br>Jan Tooropstraat 164, 1061 AE, Amsterdam

**Sponsor (in Dutch: verrichter / opdrachtgever)**	Amsterdam UMC, location VUmc,<br>De Boelelaan 1117, 1081 HV, Amsterdam
**Subsidising party**	Winclove Probiotics B.V.<br>Hulstweg 11, 1032 LB, Amsterdam<br>0031 (020) – 435 02 35<br>service@winbiotic.nl
**Independent expert (s)**	Dr. Chantal J.M. Broers<br>Amsterdam UMC, location VUmc, Amsterdam<br>Department of Pediatrics<br>CJM.Broers@vumc.nl
**Laboratory sites**	Laboratory of Amsterdam UMC, location VUmc<br>De Boelelaan 1117, 1081 HV, Amsterdam
**Pharmacy**	Pharmacy of Amsterdam UMC, location VUmc<br>De Boelelaan 1117, 1081 HV, Amsterdam

PROTOCOL SIGNATURE SHEET

Name	Signature	Date
**Head of Department:**<br>Dhr. prof. dr. H. van Goudoever<br>Head of paediatrics<br>Amsterdam UMC		**06 / 03 / 2019**
**Coordinating Investigator**<br>Dhr. dr. T.G.J. de Meij<br>Amsterdam UMC, location VUmc, Amsterdam<br>Department of Pediatric Gastroenterology		**06 / 03 / 2019**

TABLE OF CONTENTS

1. LIST OF ABBREVIATIONS AND RELEVANT DEFENITIONS 7
2. SUMMARY 9
3. ABSTRACT 11
4. INTRODUCTION AND RATIONALE 12
5. OBJECTIVES 14
6. STUDY DESIGN 14
7. STUDY POPULATION 14
   7.1 Population (base) 14
   7.2 Inclusion criteria 14
   7.3 Exclusion criteria 15
   7.4 Sample size calculation 15
8 TREATMENT OF SUBJECTS 16
   8.1 Investigational product 16
   8.2 Use of co-intervention (if applicable) 16
   8.3 Escape medication (if applicable) 17
   8.4 Intolerance to investigational product 17
9 INVESTIGATIONAL PRODUCT 17
   9.1 Name and description of investigational product(s) 17
   9.2 Summary of findings from non-clinical studies 18
   9.3 Summary of findings from clinical studies 19
   9.4 Summary of known and potential risks and benefits 20
   9.5 Description and justification of route of administration and dosage 22
   9.6 Dosages, dosage modifications and method of administration 23
   9.7 Preparation and labelling of Investigational Product 23
   9.8 Drug accountability 23
10 NON-INVESTIGATIONAL PRODUCT 24
11 METHODS 24
   11.1 Study parameters/endpoints 24
      11.1.1 Main study parameter/endpoint 24
      11.1.2 Secondary study parameters/endpoints 24
      11.1.3 Other study parameters 25
   11.2 Randomisation, blinding and treatment allocation 25
   11.3 Study procedures 27
   11.4 Withdrawal of individual subjects 28
   11.5 Specific criteria for withdrawal 31
   11.6 Replacement of individual subjects after withdrawal 31
   11.7 Follow-up of subjects withdrawn from treatment 31
   11.8 Premature termination of the study 31
12 SAFETY REPORTING 32
   12.1 Temporary halt for reasons of subject safety 33
   12.2 AEs, SAEs and SUSARs 33

12.2.1 Adverse events (AEs) ... 33
12.2.2 Serious adverse events (SAEs) ... 33
12.2.3 Suspected unexpected serious adverse reactions (SUSARs) ... 34
12.3 Annual safety report ... 34
12.4 Follow-up of adverse events ... 34
12.5 Data Safety Monitoring Board (DSMB) ... 34
13 STATISTICAL ANALYSIS ... 35
13.1 Primary study parameter(s) ... 36
13.2 Secondary study parameter(s) ... 36
13.3 Other study parameters ... 37
13.4 Interim analysis ... 37
14. ETHICAL CONSIDERATIONS ... 37
14.1 Regulation statement ... 38
14.2 Recruitment and consent ... 38
14.3 Objection by minors or incapacitated subjects ... 40
14.4 Benefits and risks assessment, group relatedness ... 40
14.5 Compensation for injury ... 40
14.6 Incentives ... 41
15 ADMINISTRATIVE ASPECTS, MONITORING AND PUBLICATION ... 41
15.1 Handling and storage of data and documents ... 41
15.2 Monitoring and Quality Assurance ... 42
15.3 Amendments ... 43
15.4 Annual progress report ... 43
15.5 Temporary halt and (prematurely) end of study report ... 43
15.6 Public disclosure and publication policy ... 43
16 STRUCTURED RISK ANALYSIS ... 44
16.1 Potential issues of concern ... 44
16.2 Synthesis ... 48
17 REFERENCES ... 52
18 APPENDIX A: CONTACT DATA PARTICIPATING CENTRES ... 55
19 APPENDIX B: WINCLOVE CERTIFICATE FOR SAFETY STATEMENT ... 57
20 APPENDIX C: SPECIFICATION OF INVESTIGATIONAL PRODUCT (Winclove 612) ... 58

1. LIST OF ABBREVIATIONS AND RELEVANT DEFENITIONS

AAD	Antibiotic Associated Diarrhoea
ABR	General Assessment and Registration form (ABR form), the application form that is required for submission to the accredited Ethics Committee; in Dutch: Algemeen Beoordelings- en Registratieformulier (ABR-formulier)
AE	Adverse Event
AISS	Amsterdam Infant Stool Scale
AR	Adverse Reaction
BSF	Bristol Stool Form
CA	Competent Authority
CCMO	Central Committee on Research Involving Human Subjects; in Dutch: Centrale Commissie Mensgebonden Onderzoek
CDAD	*Clostridium difficile*-associated diarrhoea
CDI	*Clostridium difficile* infection
CFU	Colony Forming Unit
CONSORT	Consolidated Standards of Reporting Trials
CRB	Clinical Research Bureau
CRF	Case record form
CV	Curriculum Vitae
DSMB	Data Safety Monitoring Board
ESPGHAN	European Society of Paediatric Gastroenterology, Hepatology and Nutrition
EU	European Union
EudraCT	European drug regulatory affairs Clinical Trials
FAO	Food and Agriculture Organisation
FOS	Fructo-oligosaccharides
GCP	Good Clinical Practice
GDPR	General Data Protection Regulation; in Dutch: Algemene Verordening Gegevensbescherming (AVG)
IB	Investigator's Brochure
IC	Informed Consent
IMP	Investigational Medicinal Product
IMPD	Investigational Medicinal Product Dossier
METC	Medical research ethics committee (MREC); in Dutch: medisch-ethische toetsingscommissie (METC)
MSP	Multispecies probiotic preparation
QoE	Quality of Evidence
RCT	Randomised Controlled Trial

**RR**	**Relative Risk**
**(S)AE**	**(Serious) Adverse Event**
**SPC**	**Summary of Product Characteristics; in Dutch: officiële productinformatie IB1-tekst**
**SPIRIT**	**Standard Protocol Items: Recommendations for Interventional Trials**
**Sponsor**	**The sponsor is the party that commissions the organisation or performance of the research, for example a pharmaceutical company, academic hospital, scientific organisation or investigator. A party that provides funding for a study but does not commission it is not regarded as the sponsor, but referred to as a subsidising party.**
**SUSAR**	**Suspected Unexpected Serious Adverse Reaction**
**UAVG**	**Dutch Act on Implementation of the General Data Protection Regulation; in Dutch: Uitvoeringswet AVG**
**WHO**	**World Health Organisation**
**WMO**	**Medical Research Involving Human Subjects Act; in Dutch: Wet Medisch-wetenschappelijk Onderzoek met Mensen**

2. SUMMARY

Rationale: Antibiotic Associated-Diarrhoea (AAD) is a common complication of antibiotic treatment in children. Certain probiotic strains are proven to be effective in reducing the risk on AAD, however, there is not enough evidence to recommend use of any of the multispecies probiotic preparations (MSP). Winclove 612 (Winclove Probiotics B.V., the Netherlands), the MSP used in this trial, is a preparation consisting of 8 probiotic strains. In one placebo-controlled randomized clinical trial with a comparable preparation in a group of 41 healthy adults receiving antibiotics, the group receiving the probiotic had significantly reduced rate of diarrhoea-like bowel movements. To date, no randomized controlled trials have been performed in larger groups or in children to assess effectiveness of the preparation in the prevention of AAD.

Objective: The aim of the study is to assess the efficacy and safety of a multispecies-strain probiotic in the prevention of AAD in children.

Study design: The study is an international, multicentre, randomized, double-blind, placebo-controlled, parallel group trial with an allocation ratio of 1:1, performed in Poland and the Netherlands.

Study population: In total 350 children receiving broad-spectrum antibiotics who are otherwise healthy, will be included. Of them, the aim is to include half of the population (180 children) in the Netherlands, more specifically in AUMC and OLVG.

Intervention: The participants will be randomly assigned to one of two subgroups in which they will receive either the MSP at a dose of 10^{10} CFU per day in two doses or a placebo product, starting at the same day as antibiotics are started, and continuing use for one additional week after cessation of antibiotic use (up to maximum 17 days). The MSP used in this study is called Winclove 612 (Winclove Probiotics B.V., the Netherlands).

Main study parameters/endpoints: The primary outcome measure will be the incidence of AAD. AAD will be defined as three or more loose or watery stool (a score of A on the AISS or 5-7 on the BSF scale) per day in a 24-hour period, either caused by *C. difficile* infection or of otherwise unexplained aetiology (after testing for common diarrhoeal pathogens), occurring during the intervention period.

Nature and extent of the burden and risks associated with participation, benefit and group relatedness: Participants will use the investigational product for a maximum of seventeen days. The use of the investigational product is considered to be safe in children. There is a chance on side effects, however side effect are mild gastro-intestinal symptoms such as bloating and flatulence and will disappear in a couple days.

Besides using the investigational product, all participants need to collect stool samples at 6 times during. In case of diarrhoea during the intervention period or the following seven days, we ask for one more stool samples. Furthermore, a diary has

to be kept where participants log the consistency of their stool during the intervention period and the seven following days.
No extra (invasive) physical examinations or other tests are needed, nor are participants required extra site visits to the study site.
Participation in this trial could potentially benefit the patients in the probiotics-group. We hypothesize that probiotics can restore dysbiosis caused by antibiotics and consequently prevent AAD. Patients randomly assigned to the placebo-group will receive standard of care and will not benefit from participating in this study. The knowledge obtained about the effects of probiotics could potentially benefit future patients receiving antibiotics.

3. ABSTRACT

Background

Antibiotic Associated-Diarrhoea (AAD) is a common complication of antibiotic treatment in children. Certain probiotic strains are proven to be effective in reducing risk of AAD, however, there is not enough evidence to recommend use of any of the multispecies probiotic preparations. Winclove 612 is a preparation consisting of 8 probiotic species. Two studies in patients receiving antibiotics demonstrated a positive effect of probiotics on the incidence of AAD. A placebo-controlled randomized clinical trial with a comparable preparation in a group of 41 healthy adults receiving antibiotics confirmed these findings; the group receiving probiotics had a significantly reduced rate of diarrhoea-like bowel movements. To date, no randomized controlled trials have been performed in larger groups or in children to assess effectiveness of the preparation in the prevention of AAD.

Aim

The aim of the study is to assess the efficacy and safety of this multispecies probiotic in the prevention of AAD in children.

Method:

350 participants will be randomly assigned to one of two subgroups in which they will receive either a multispecies probiotic at a dose of 10^{10} CFU per day in two doses or a placebo product, starting at the same day as antibiotics are started, and continuing use for one additional week after cessation of antibiotic use (up to maximum 17 days). Primary outcome will be the incidence of AAD.

Discussion

Since no trials have assessed the effectiveness of this specific formulation in the prevention of AAD in children, results of this trial may contribute to the development of future guidelines. Several issues, like uncertain AAD incidence or reluctance of caregivers to consent for the study, may negatively influence it’s course. however, certain adjustments were made to overcome or prevent these problems.

4. INTRODUCTION AND RATIONALE

Antibiotics are well known to cause disturbances in the composition of the intestinal microbiota, leading to the development of gastrointestinal (GI) symptoms (1). Antibiotic-associated diarrhoea (AAD), defined as diarrhoea that occurs in relation to antibiotic treatment with the exclusion of other causes, is a common complication of antibiotic use in children (2). In a Cochrane meta-analysis, published in 2015, the pooled risk of AAD in children was 19% (3). However, this risk varied greatly between individual studies, ranging from 2.1% (4) to 80% (5), depending on factors such as the adopted definition of diarrhoea, the study population and the type of antibiotic treatment (6). According to the World Health Organisation (WHO), diarrhoea is defined as passage of loose or liquid stools usually at least three times per day, or a more frequent passage than is normal for the individual (7). In small children, especially in infants, a change in stool consistency is more important in the definition of diarrhoea rather than the number of stool movements (8), hence the applied diagnostic criteria for diarrhoea differ from each other in paediatric population studies. The pathogenesis of AAD is not fully understood. It may be caused by a specific enteric pathogen (e.g., *Clostridium difficile*, *Clostridium perfringens*, *Staphylococcus aureus*, *Candida albicans*), metabolic consequences of altered intestinal microbiota or a direct effect of antibiotics on the mucosa (9). AAD may vary both in severity (from uncomplicated diarrhoea to pseudomembranous colitis) and in incubation time (from the first day of antibiotic treatment to 8 weeks after discontinuation) (10). The impact of antibiotics on the abundance of commensal micro-organisms in the gut underlines the hypothesis that administration of probiotics could reduce the incidence of AAD.

Probiotics are 'live micro-organisms that, when administered in adequate amounts, confer a health benefit on the host' (11). Data from earlier studies suggest that doses of $>5\times10^9$ colony forming units (CFU) of probiotic micro-organisms are more effective than doses $<5\times10^9$ CFU in preventing AAD (12). According to the European Society of Paediatric Gastroenterology, Hepatology and Nutrition (ESPGHAN) and FAO/WHO, probiotic properties are species-specific and strain-specific, so each strain or their combinations should be examined separately (2, 13). There is presumptive evidence for the effect of probiotics in prohibiting AAD. However, probiotic preparations consisting of more than one strain, multispecies probiotic preparations (MSP), are not yet routinely recommended to reduce the incidence of AAD in children, because sufficient evidence is still lacking (2). Nonetheless, there are certain studies focusing on other strains and combinations of strains, suggesting evidence of their effectiveness (3, 14). *Saccharomyces boulardii* and *Lactobacillus rhamnosus* GG are single probiotic strains proven to be effective in the prevention of AAD (15, 16). In the international ESPGHAN protocol these probiotics are considered safe for use in otherwise healthy populations and can be considered using for preventing AAD in children. Results from these studies have resulted in the ESPGHAN recommending *L. rhamnosus GG*

(moderate quality of evidence (QoE), strong recommendation) and *S. boulardii* (moderate QoE, strong recommendation) for preventing AAD in children if the use of probiotics is considered. In case of *Clostridium difficile*-associated diarrhoea (CDAD), it is recommended to use *S. boulardii* (low QoE, conditional recommendation) (2).

The investigational product used in this study is a MSP called Winclove 612. There are no studies yet investigating the effectiveness of this specific MSP. However, some studies have been performed with Ecologic AAD, a very resembling MSP. Ecologic AAD is a preparation consisting of nine different probiotic strains, including two strains of *Bififobacterium*, six strains of *Lactobacillus* and *Enterococcus faecium* W54. Winclove 612 has a similar composition to Ecologic AAD. However, in contrast to Ecologic AAD, Winclove 612 does not contain *Enterococcus faecium* W54. The species *E. faecium* is not recommended for use in children by the European Society for Paediatric Gastroenterology Hepatology and Nutrition (ESPGHAN) due to unclear safety issues (17) and, therefore is excluded from the current used formulation.
In one RCT conducted in 41 healthy adult volunteers receiving amoxicillin with either Ecologic AAD or placebo, subjects in the experimental group had a significantly lower rate of diarrhoea-like bowel movements compared with those in the placebo group (48% vs 79%, respectively, relative risk (RR)=0.61, p<0.05) (18). Another RCT conducted in 45 adult patients with a chronic obstructive pulmonary disease exacerbation who were treated with antibiotics did not reveal a difference in the rate of diarrhoea-like bowel movements between the Ecologic AAD and placebo groups (77% vs 70%, respectively, RR=1.1, p>0.05)(19). However, this study was carried out in a very specific group of patients, consisting of subjects with a history of frequent and prolonged antibiotic use. In another cohort study with 199 patients receiving probiotics next to their antibiotic treatment the incidence of AAD was 0,5%. This is much lower than the expected incidence of 5-39% in that study population suggesting a beneficial role of Ecologic AAD (20). In a retrospective case report series of 10 *Clostridium difficile* infection (CDI) patients, 5 of whom experienced recurrent CDI, all patients received, besides antibiotics before the treatment of CDI, two times daily Ecologic AAD, which resulted in complete recovery (21).
So far, there have been no RCTs using Winclove 612 or Ecologic AAD carried out in larger groups of participants or in children.

This will be the first randomized trial evaluating the efficacy of a probiotic preparation with the above-mentioned composition in the prevention of AAD in children. AAD is a frequent complication of treatment that extends the stay of patients in the hospital, requires medical checks and increases the costs incurred by healthcare centres and patients. Since no multispecies probiotic is currently recommended for the prevention of AAD, this study has a chance to influence the shape of official guidelines. The study will be conducted in accordance with the applicable clinical trial rules (registration of the protocol in the clinicaltrials.gov registry, publication of a report prepared in

accordance with the SPIRIT statement, reporting in accordance with the CONSORT statement), which further increases the chance of including its results in systematic reviews and induce alterations of current guidelines.

5. OBJECTIVES

Study aim

The primary study objective of the study is to assess the effectiveness of a specific probiotic preparation (Winclove 612 (Winclove Probiotics, Amsterdam, the Netherlands)) in reducing the incidence of AAD in children (see chapter 11.1.1). Secondary outcomes will include AAD based on other definitions, duration of diarrhoea, hospitalization and adverse events and the influence on intestinal microbiota composition (for a more detailed description of secondary outcomes, see chapter 11.1.2).

6. STUDY DESIGN

Trial design

The study is an international, multicentre, randomized, double-blind, placebo-controlled, parallel group trial with an allocation ratio of 1:1, performed in Poland and the Netherlands. The study protocol has already been approved by the bio-ethical commission of the Medical University of Warsaw. This study protocol is available online (22) and is registered at clinicaltrials.gov (NCT03334604).

7. STUDY POPULATION

7.1 Population (base)

Hundred seventy-five (175) participants in this study will be recruited among both the inpatient and outpatient clinics from the department of Paediatrics at the Amsterdam UMC, locations AMC and VUmc (Amsterdam) and the Onze Lieve Vouwe Gasthuis, location Oost and West (Amsterdam) and via pharmacies. Other hospitals and medical care centres would also be plausible sources of participants, providing the presence of adequately trained personnel and depending on number of inclusions, this will be evaluated after 6 months.

The recruited participants will all be children with an age between 3 months and 18 years old. The included children have to be receiving antibiotics, but should otherwise be healthy.

Hundred seventy-five (175) other participants necessary for this study will be included from the ongoing trial at the Medical University of Warsaw.

7.2 Inclusion criteria

In order to be eligible to participate in this study, a subject must meet all of the following criteria:

- Age between 3 months and 18 years old
- Therapy with oral or intravenous antibiotics for common infections
- Ability to start the probiotic intervention within 24 hours after the start of antibiotic intake
- Use of broad-spectrum antibiotics
- Signed informed consent

7.3 Exclusion criteria

A potential subject who meets any of the following criteria will be excluded from participation in this study:

- Use of antibiotics or probiotics in the last 4 weeks
- Presence of a severe or generalised infection
- History of pre-existing diarrhoea within the previous 4 weeks
- Exclusive breastfeeding
- Tube feeding
- Use of proton-pump inhibitors, laxatives or antidiarrheal drugs during the last 2 weeks or during the intervention period
- History of severe chronic disease (e.g.; cancer, inflammatory bowel disease, tuberculosis)
- Critical or life-threatening illness, including systemic symptoms of sepsis, pancreatitis, multi-organ failure or admission to intensive care unit
- Immunodeficiency
- Allergy or hypersensitivity to (ingredients) of the intervention product
- Abdominal surgery in the last month
- If it can reasonably be expected that the patient will go for an abdominal surgery within a month (for example patients with an appendicitis initially treated with antibiotics)

7.4 Sample size calculation

The pooled risk of AAD as a consequence of using broad-spectrum antibiotics, determined from previous studies conducted at the Medical University of Warsaw (23-25) is 13.5%. This is less than the incidence of 19% from a Cochrane meta-analysis on the protective effect of probiotics on AAD (3). There are no well documented data on the incidence of AAD in the Netherlands. We have chosen to perform a sample size calculation based on an expected AAD risk of 16%. To show a difference of 11% in the treatment effect in the study groups with α=0.05% and 80% power, and assuming a 20% withdrawal rate, a total of 350 participants will be needed. Sample size calculations were performed with StatsDirect (V.3.1.4, StatsDirect statistical software; StatsDirect, Chesire, UK) (22).

8 TREATMENT OF SUBJECTS

Interventions

The participants will be randomly assigned to one of the two subgroups in which they will receive either the MSP at a dose of 10^{10} CFU per day in two doses or a placebo product. The MSP used in this study (Winclove 612) contains the following eight strains:

- *Bifidobacterium bifidum W23*
- *Bifidobacterium lactis W51*
- *Lactobacillus acidophilus W37*
- *Lactobacillus acidophilus W55*
- *Lactobacillus paracasei W20*
- *Lactobacillus plantarum W62*
- *Lactobacillus rhamnosus W71*
- *Lactobacillus salivarius W24*

Those strains are based on a carrier material consisting of maize starch, maltodextrins, fructo-oligosaccharides (FOS) P6, maize dextrin P9, mineral mix (potassium chloride, magnesium sulphate, manganese sulphate), hydrolysed rice protein and enzymes (amylase). The control group will receive a placebo product (Winclove Probiotics, Amsterdam, the Netherlands) that is indistinguishable in colour, smell and taste from the used MSP. Both the MSP and placebo are a powder, which have to be dissolved in water or milk before use. The interval between antibiotic intake and probiotic consumption has to be at least two hours. Both study products (MSP and placebo) will be manufactured and supplied free of charge by Winclove Probiotics B.V. (Amsterdam, the Netherlands).

8.1 Investigational product

The intervention used in this study will be a food supplement; the MSP, Winclove 612 (for more detailed information about the MSP, see section 9). The control group will receive a placebo.

The role of the producer of the investigational product in the course of the study

The producer of the product had the opportunity to comment on the first draft of the protocol, but any final decisions regarding its shape were made by the research team. The producer will have no influence on the course of the study as well as the analysis and interpretation of the data. The results of the study will be published, regardless of whether they are positive or negative.

8.2 Use of co-intervention (if applicable)

Not applicable.

8.3 Escape medication (if applicable)

Not applicable.

8.4 Intolerance to investigational product

Intolerance to the investigational product is unlikely. Participants with a known allergy or hypersensitivity to (ingredients) of the study product will be excluded. However if patients are intolerant, they will be treated according to physicians discretion. Study follow-up will continue according to the intention-to-treat principle.

9 INVESTIGATIONAL PRODUCT

9.1 Name and description of investigational product(s)

The investigational product used in this study will be a MSP called Winclove 612. It is a multispecies probiotic formulation, developed to prevent and restore antibiotic-induced disturbances of the microbiota and subsequently the risk of antibiotic associated side effects. Together with the Maastricht University Medical Centre (MUMC+) in the Netherlands, Winclove Probiotics has selected the strains from the commercially available probiotic strain collection of Winclove Probiotics based on their capacity to restore the intestinal microbiota. More specifically, they were selected on their capacity to inhibit *Clostridium difficile* and its toxins, being one of the most important factors in development of AAD/CDAD, their inhibition of *Candida* spp. and their *in vitro* survival of the GI-tract to reach the gut in proper state.

The formulation consists of 8 specifically selected probiotic strains: *Bifidobacterium bifidum* W23, *Bifidobacterium lactis* W51, *Lactobacillus acidophilus* W37, *Lactobacillus acidophilus* W55, *Lactobacillus paracasei* W20, *Lactobacillus plantarum* W62, *Lactobacillus rhamnosus* W71 and *Lactobacillus salivarius* W57 and is based on a carrier material consisting of maize starch, maltodextrins, amylases, fructo-oligosaccharides (FOS) P6, maize dextrin P9, mineral mix (potassium chloride, magnesium sulphate and manganese sulphate), vanilla flavor and vegetable protein. The mixture is 2 years stable at room temperature, no refrigeration needed.

There are no studies yet investigating the effectiveness of this specific MSP. However, some studies have been performed with Ecologic AAD, a very comparable product. Ecologic AAD is a preparation consisting of nine different probiotic strains. It is available in many countries, and on the Dutch market under the brand name Winbiotic Pro.AD. Ecologic AAD consists of the exactly the same trains as Winclove 612. However, in contrast to Winclove 612 it does contain *Enterococcus faecium* W54 too. The species *E. faecium* is not recommended for use in children by the European Society for Paediatric Gastroenterology Hepatology and Nutrition (ESPGHAN) due to unclear safety issues (17) and, therefore is excluded from our current used formulation. It is

very plausible to presume that effects found for Ecologic AAD will apply to Winclove 612 too. The specification of the investigational product can be found in Appendix C.

9.2 Summary of findings from non-clinical studies

Selection criteria for development of Winclove 612 were to screen the probiotic bacteria on their ability to inhibit the pathogens *Clostridium perfringens, Enterococcus faecalis, Escherichia coli* and *Bacillus subtilus*. Pathogen inhibition is measured *in vitro* using the well diffusion test, described by Hechard (26). Briefly, a nutritious medium is poured into a petri-dish. In this agar medium a potentially pathogenic organism is grown. The probiotic strain is added to the hole (8 mm diameter) in the agar. If the probiotic strain inhibits the pathogen, a clear zone is shown around the hole. The diameter of this clear zone is measured. The larger this zone, the better is the capacity of the probiotic strain to inhibit the pathogenic organism. It can be concluded that most strains in Winclove 612 are very capable to inhibit one or several of the above mentioned pathogens.

It has been shown that *Clostridium difficile* is the cause of diarrhoea in 20% of AAD. Moreover *Clostridium difficile* associated diarrhoea (CDAD) can lead to severe and life threatening pseudomembranous colitis. Therefore inhibition of *Clostridium difficile* growth was examined *in vitro* using a co-culture method. Growth of *C. difficile* was compared to growth of the strain in the presence of a probiotic strain. The effect of the probiotic bacteria on the production of the toxins A and B by *C. difficile* was measured, using an ELISA assay. Results of the analyses are shown in figure 1.

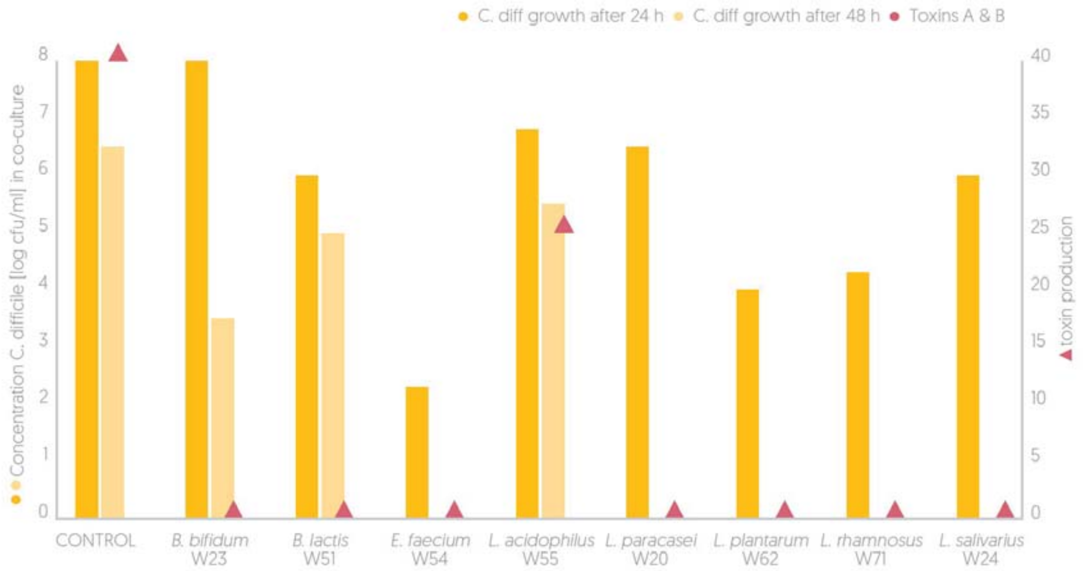

Figure 1: Inhibition of growth of C. difficile over time (after 24hr and 48hr) and shows that several strains completely inhibit the growth of C. difficile, especially after 48h of co-culture. Furthermore, it can be seen that 8 strains inhibit the production of toxins A&B (red triangles).

9.3 Summary of findings from clinical studies

A double-blind, placebo controlled randomized controlled trial showed that Ecologic AAD is able to significantly reduce the risk of diarrhoea-like defecation (18). The study was executed in the Maastricht University Medical Centre (MUMC+) in the Netherlands, where 41 healthy volunteers were given 500 mg of the antibiotic amoxicillin twice daily for 7 days and were randomized to either 5 gram Ecologic AAD or placebo, twice daily for 14 days.
Bowel movements with a frequency ≥3 per day for at least 2 days and/or a consistency ≥5 for at least 2 days, were reported less frequently in the probiotic compared to the placebo group (48% vs 79% ($p<0.05$)). Mean number of enterococci increased significantly from log 4.1 at day 0 to log 5.8 (day 7) and log 6.9 (day 14) CFU/gram faeces ($p<0.05$) during probiotic intake. Apart from an increase in enterococci no significant differences in microbial composition and metabolic activity were observed in the probiotic compared with the placebo group. However, changes over time were present in both groups, which differed significantly between the probiotic and the placebo arm, suggesting that the amoxicillin effect was modulated by probiotic intake.
The effect of Ecologic AAD was also investigated in COPD patients, being a target group with a history of frequent antibiotic use (19). In this randomized placebo-controlled double-blind study 30 COPD patients treated with antibiotics for a respiratory tract infection received Ecologic AAD twice daily for two weeks. During and after antibiotic treatment, DGGE-based similarity indices (SIs) were high (≥84%) and band richness was relatively low both remaining stable over time. No difference in SIs was observed between patients with and without diarrhea-like bowel movements. Ecologic AAD had a modest effect on the bacterial subgroups. Nevertheless, it did not affect the composition of the dominant faecal microbiota nor the occurrence of diarrhea-like bowel movements. The dominant faecal microbiota was not affected by antibiotics in this COPD population, suggesting an existing imbalance of the microbiota, which may also have contributed to the lack of effect by probiotic intake.

In a cohort in Austria with 199 people surgery patients were -after an antibiotic regimen- longitudinally treated with Ecologic AAD (20). In this study, 199 patients of the surgery ward of Landesklinikum Thermenregion Neunkirchen (Austria) received Ecologic AAD next to antibiotic treatment, twice daily (total $1*10^{10}$ CFU/day), starting from the first day of antibiotic treatment and until one week after cessation of antibiotics. During the test period only 2 of the 199 patients developed AAD, which probably had been caused by overgrowth of *Clostridium difficile* in one of the patients. Based on the knowledge from literature and experiences in the Landesklinikum the incidence of AAD ranges from 5-39% in patients using

antibiotics, in this study one might expect 10-80 patients suffering from AAD. An observed almost complete absence of AAD incidence (0.5%) in this cohort of Ecologic AAD treated patients strongly suggests a beneficial role of Ecologic AAD therapy during and after antibiotic treatment.

In a retrospective case report series of 10 *Clostridium difficile* infection (CDI) patients, 5 of whom experienced recurrent CDI, all patients received, besides antibiotics before the treatment of CDI, two times daily Ecologic AAD, which resulted in complete recovery. Moreover no adverse events were reported (21).

In their global guidelines, the World Gastroenterology Organisation (WGO) evaluates pro- and prebiotics on their efficacy and safety. In this guideline Ecologic AAD is evaluated as *a randomized trial with dramatic effect in the prevention of antibiotic-associated diarrhea (27)*. More specifically, the evidence for Winclove's probiotic formulation Ecologic AAD is evaluated as level 2 evidence: Randomized trial or observational study with dramatic effect. The recommendation of Ecologic AAD as an effective and safe probiotic for reducing antibiotic-associated diarrhea by the WGO is very promising in implementing probiotics as a standard prescription next to antibiotics.

It is very plausible to presume that effects found for Ecologic AAD will apply to Winclove 612 too as the former consists of all eight strains that can be found in Winclove 612 too. The only strain that is included in Ecologic AAD that is not present in Winclove 612 is *E. faecium* since it is not recommended for use in children by the ESPGHAN due to unclear safety issues. Consequently, we don't expect more or other side effects from Winclove 612 then are described for Ecologic AAD.

9.4 Summary of known and potential risks and benefits

Based on all clinical trials performed with Ecologic AAD (18-21, 28), patients can expect that by using Winclove 612 less diarrhoea-like bowel movements will occur, and microbiota is restored or even prevent dysbiosis.

The potential risks of participation are low, especially as probiotic intervention is started after bacteraemia and sepsis are excluded. No serious events are expected from administration of the test product or control product. If adverse effects occur, they are likely to be minor gastro-intestinal symptoms, such as bloating or flatulence, especially when one uses probiotics for the first time. These effects are expected to disappear after a few days.

Recently the Netherlands Organization for Applied Scientific Research (in Dutch: de Nederlandse Organisatie voor toegepast-natuurwetenschappelijk onderzoek; TNO) published a practical guide for the use of probiotics for the prevention of antibiotic-

associated diarrhea (29). According to the Agency for Healthcare Research and Quality (AHRQ) there is not enough information to confidently judge the safety of probiotic-based interventions. Still, probiotic products are generally regarded as safe, and they are used both by healthy and ill people globally. Possible safety concerns are especially concerning for patients with a weakened or compromised immune system. For that reason those children are being excluded from current study. The the multi-strain formulation of *Lactobacillus rhamnosus*, *Lactobacillus acidophilus*, *Bifidobacterium lactis,* which all are present in our investigational product, was found to have a positive effect on the incidence of AAD. Furthermore, a one-star recommendation (an effect shown in only one study, a trend supported by two or more studies, or the presence of a strain that satisfies one of the above criteria (showing an effect in one study or a trend in at least 2 studies) in sufficient amounts in food supplement or dairy product with a mixed formulation) was assigned to formulations that showed a trend supported by two or more studies, including *Bifidobacterium bifidum*, *Bifidobacterium lactis*, *Lactobacillus acidophilus*, *Lactobacillus paracasei*, *Lactobacillus plantarum*, *Lactobacillus rhamnosus*, *Lactobacillus salivarium* (all strains in our product) with a minimal daily dose of 10 billion CFU. One of the multispecies probiotic products they recommend is Winbiotic Pro-AD (containing all 8 strains of our investigational product plus *Enterococcus faecium W54*) in a dose of $1x10^{10}$ CFU/day.

Even though most literature and meta-analysis (30, 31) show that probiotics are safe and effective in preventing AAD, there are some studies questioning this. They state that most studies on probiotics do not report adverse events or possible harms well(32, 33). One of these trials examined the effects of multi-strain probiotics on post-antibiotic reconstitution of the murine and human mucosal microbiome niche (34). Compared to spontaneous post-antibiotic recovery, probiotics induced a markedly delayed and persistently incomplete indigenous stool/mucosal microbiome reconstitution and host transcriptome recovery toward homeostatic configuration. This underlines the hypothesis that probiotics, if wrong species are administrated, can be counterproductive.
There are concerns by some researchers that probiotics may cause infections, especially in critically ill or immunocompromised patients. However, there are very few case reports describing these infections and studies showing an absence of any change in the prevalence of probiotic induced bacteremia (35). There is a theoretical possibility of lateral gene transfer between probiotic organisms and other organisms in the gut or other site, however no clinical evidence of transfer of antimicrobial resistance has ever been seen (35).

9.5 Description and justification of route of administration and dosage

The product has a concentration of 2.5*10^9 CFU/gram with an advised daily dosage of 2 grams twice daily (so 10^{10} CFU per day). The dosage of Winclove 612 is in accordance with most probiotic dosages used in human studies and many commercially available products, ranging from 10^9-10^{10} CFU/dose (K6. Probiotica verkrijgbaar in Nederland demonstrates a list of all probiotics available in the Netherlands). It is not possible to state a general dose for probiotics; some have shown to be effective at lower levels, while other require substantially more. The dosage of Winclove 612 in this study is based on prior human studies in healthy volunteers, COPD patients and general surgery patients with the product (18-21, 28) showing a health benefit without adverse reactions.
The concentration of probiotics needed to obtain a clinical effect is often quoted as $\geq 10^6$ colony-forming units/ml (CFU) in the small bowel and $\geq 10^8$ CFU/g in the colon (36). Dose–response studies are, however, scarce, and it is not known whether the percentage survival is stable for various ingested doses.
The survival of probiotics depends on their intrinsic resistance, on host factors and on the vehicle in which they are ingested. The main obstacles to survival being gastric acidity and the action of bile salts. Well-controlled, small-scale studies on diarrhea in both adults and infants have shown that probiotics survive in sufficient numbers to affect gut microbial metabolism (37). Survival rates have been estimated at 20–40% for selected strains, but differ between strains (38). Approximately 1–10% of *Lactobacillus acidophilus* ingested in fermented products was found to survive until the ileum in several human studies using intestinal intubation techniques (39). In contrast, 30% of ingested *Bifidobacterium* was found in the colon (40). Other studies in healthy volunteers with different probiotic preparations showed that the faecal concentrations of ingested *Lactobacillus acidophilus*, *Lactobacillus salivarius* and *Lactobacillus rhamnosus* strains reached around 10^6 CFU/g. Probiotics are usually excreted within a few days of their ingestion in faeces at the same rate as or even more quickly than a transit marker (37).
The Cochrane review, published the 30th of April 2019, on prevention of pediatric AAD states that studies using a dose of >5x10^9 (n=20) showed a significant reduction of AAD, whereas studies using a dosage of <5x10^9 (n=12) did not. These studies included children from all 3 days old up to 17 years of age. (41, 42). Despite proven to be safe in these dosage, dose-response studies for AAD and on the microbiome are missing. A dose of 1x10^{10} is generally accepted in studies with children and this study will investigate whether this dose has the same effect on the incidence of AAD and the microbiota of young children compared to older children.
In the supplementary file (K6. Probiotica verkrijgbaar in Nederland) all probiotics available in The Netherlands are listed. As shown, most are recommended in a daily dose of ≥ 10 billion (i.e. 1x10^{10} CFU or 10x10^9 CFU).
With this study we want to contribute to the knowledge on response to probiotics in children from different ages. By performing sub analyses we want to see whether the

microbiota of young children is affected in the same amount as the microbiota of older children.

9.6 Dosages, dosage modifications and method of administration

Each sachet contains 2 grams of probiotics with $2.5*10^9$ CFU per gram. Participants need to take 2 sachets per day; one in the morning and one in the evening (so 10^{10} CFU per day). The content of a sachet needs to be dissolved in 100mL luke-warm water, milk or yoghurt, and not in fruit juices or hot liquids. When antibiotics are administered orally, the investigational product needs to be taken at least two hours prior to or two hours after the use of the antibiotics. Furthermore, the solution containing probiotics needs to be ingested orally, preferably on an empty stomach. It is recommended not to use disinfectant mouthwash prior to ingestion. product

9.7 Preparation and labelling of Investigational Product

Preparation and labelling will be conducted at Winclove Probiotics B.V. In the Netherlands and European Union all probiotics are considered to be food supplements and therefore have to be produced under HACCP regulations, the Dutch regulation system for safety and hygiene in food and food supplements. Winclove has been producing multispecies probiotics for over 25 years. Winclove's ISO 22000:2005 certificate for safety statement can be found in appendix B.
Products will be transported to the experimental pharmacy of the Amsterdam UMC, location VUmc where the products will be stored and distributed to pharmacies of the participating centers.

9.8 Drug accountability

The boxes of probiotics will be given to subjects by the coordinating investigator (supply for the complete project period). Boxes include probiotic sachets and Winclove's user instruction. The shipment, coded blank boxes and coded sachets, will be delivered at the pharmacy of Amsterdam UMC, location VUmc and AMC and OLVG location Oost and West by courier. The food supplements will be safely stored and locked by key at the pharmacies and at one external location on each site outside of the pharmacies on the paediatric ward and will only be accessible by the research team and involved pharmacists. The product will be kept at room temperature. The coordinating investigator and/or pharmacy will distribute the intervention (placebo or probiotic mixture) to participants in person. Patients are supposed to use all sachets provided. For compliance reasons, the patients will be asked to save all the empty sachets, and bring everything (used and unused sachets) in return to the investigator. After counting the empty sachets, to get numbers on compliance, all study material can be destroyed. As this is a food supplement, there is no need for special waste treatment.

The investigational product (Winclove 612) and the placebo product will be produced free of charge by Winclove Probiotics B.V.

10 NON-INVESTIGATIONAL PRODUCT

The placebo is indistinguishable in color, smell and taste from the investigational product. It contains maize starch, maltodextrin, potassium chloride, hydrolysed rice protein, magnesium sulphate, amylase and manganese sulphate. The specification of the products (the investigational product and placebo) can be found in Appendix C.

11 METHODS

11.1 Study parameters/endpoints

11.1.1 Main study parameter/endpoint

The primary outcome measure will be the incidence of AAD. AAD will be defined as three or more loose or watery stools (a score of A on the AISS or 5-7 on the BSF scale) per day in a 24-hour period, either caused by *C. difficile* infection or of otherwise unexplained aetiology (after testing for common diarrhoeal pathogens), occurring during the intervention period.

11.1.2 Secondary study parameters/endpoints

Secondary outcomes assessed during the intervention period will include AAD based on two other definitions of diarrhoea used in previous studies:

- Three or more loose or watery stools per day (defined as above) for a minimum of 48-hour period either caused by *C. difficile* infection or of otherwise unexplained aetiology
- Two or more loose or watery stools per day (defined as above) for a minimum of a 24-hour period either caused by *C. difficile* infection or of otherwise unexplained aetiology

Other secondary outcome measures will be as follows:

- The duration of diarrhoea (defined as the time until the normalisation of stool consistency according to the BSF or AISS scale (on BSF scale, numbers 1, 2, 3 and 4; on AISS scale, letters B, C or D), and the presence of normal stools for 48 hours)
- Incidence of any diarrhoea (three or more loose or watery stools (defined as above)) per day, for a minimum of 24 hours, regardless of its aetiology.
- *C. difficile*-associated diarrhoea (diarrhoea defined as in the primary outcome but caused by *C. difficile* confirmed by the presence of toxin-producing *C. difficile* in stools (positive toxin tests))
- Discontinuation of the antibiotic treatment due to severity of diarrhoea

- Hospitalisation caused by diarrhoea in outpatients
- Need for intravenous rehydration in any of the study groups
- Adverse events
- Intestinal microbiota composition, composition of both subgroups will be compared
- Amino acid/metabolomics analysis
- Effects of different types of antibiotics on the incidence of AAD
- Effects of different types of antibiotics on the incidence of the microbiome

11.1.3 Other study parameters

Baseline characteristics and other study parameters important for this study will be obtained at inclusion. These values and parameters will include:

- Standard demographic data
- Age and date of birth
- Gender
- Type of antibiotic used during intervention period
- If applicable: feeding type (breast feeding, formula or combination)
- Use of antibiotics in last 4 weeks
- History of diarrhoea during last 4 weeks
- Use of antibiotics, probiotics, proton pump inhibitors, laxatives, immunosuppressive or antidiarrheal drugs during the last 4 weeks
- Use of other medication during last 4 weeks
- History of chronic disease
- History of gastro intestinal surgery and gastro-intestinal anomalies
- Inflammatory bowel disease, coeliac disease, diabetes mellitus type I/II
- (History of) critical or life-threatening illness
- Immunodeficiency
- Allergy or hypersensitivity to (ingredients of) the intervention products
- Presence of other comorbidities
- Medical history

11.2 Randomisation, blinding and treatment allocation

Randomization and allocation concealment

The randomisation and coding will be performed centrally by Winclove Probiotics B.V. by a subject not involved in the study using a computerised programme. Blocked randomisation (blocks of four) will be used to ensure a good balance of participants' characteristics in each group. Allocation will be determined by using a computerised random number generation process. All study products will be sequentially numbered. Coded study products will be handed over to the researchers. When the study has ended, participants will be divided into two blinded groups, which will be used in the statistical analysis. After performing the analyses, code numbers will be opened by the

coordinating and principal investigator. Sealed envelopes containing the allocation of each number will be handed to the principal investigator ensuring that if a medical problem or emergency occurs for which the treating physician needs to know the treatment allocation (probiotics or placebo), the code can at all times be broken.

Agreements are made with the manager of the experimental medicine pharmacy (dr. I. Bartelink, pharmacist) and team leader of the experimental medicine pharmacy (A. Admiraal) about the randomisation, storage and distribution of the product. Since the pharmacy of the Amsterdam UMC location VUmc does not have enough storage capacity to store all product and divide them to the participating centres, we decided in joint agreement that randomisation and distribution to participating centres will be done centrally at Winclove Probiotics B.V. as described previously. Winclove Probiotics B.V. will manage this randomisation list and will make a sealed envelope for each randomisation number with information for whether this randomisation number corresponds with either probiotics or placebo. These sealed envelopes will all be put in a bigger sealed envelope. These sealed randomisation codes will be send to and stored in the Amsterdam UMC location VUmc, so the code can be broken 24/7 in case of a medical emergency.
After randomisation, Winclove Probiotics B.V. will deliver the randomised and coded study products to the (experimental) pharmacy of the Amsterdam UMC location VUmc and pharmacies of participating centres. Products will be stored here and handed out to participants when they are being included. A part of the products will be stored outside of the pharmacy (on the paediatric ward or E.R.) to make sure participants can be included when the (experimental) pharmacy is closed. The pharmacy will make sure the products are stored in a correct way by performing audits etc. The products will be stored in boxes containing 34 sachets of the investigational product or placebo. The person handing out the products will be aware of the study protocol and how much the participants need to use (2 sachets per day during the antibiotic treatment plus the 7 following days, with a maximum of 17 days (and so a maximum of 34 sachets)). The pharmacist or person handing out the products can give additional information on how to use the products and can remind participants to return the empty used sachets and the full unused sachets afterwards to the pharmacy to verify the compliance.

Blinding

The probiotic preparation and placebo will be stored in identical packages. The contents will look, smell and taste the same. Researchers, caregivers, participants, medical personnel and outcome assessors will all be blinded to the intervention until the study is completed and the data analysed.

11.3 Study procedures

A total of 350 children aged 3 months - 18 years, undergoing antibiotic treatment for common infections, will be enrolled in this double-blind, placebo controlled trial. After obtaining written consent to participate in the study, the children will be randomized to receive either Winclove in a dose of 10^{10} CFU daily (2 gram of probiotics with $2.5x10^{9}$ CFU per gram twice per day), or an identical placebo. The product will be used for the duration of antibiotic treatment and the 7 following days with a maximum of 17 days (*the intervention period*). The primary outcome measures will include incidence of AAD during intervention period and 7-day follow-up period. During this time, frequency of bowel movements and consistency based on the Amsterdam Infant Stool Scale (AISS) (43) for children younger than one year and Bristol Stool Form (BSF) scale (44) for children older than one year will be recorded in a study diary, which will be handed out to the participants after recruitment. A score of 'A' on the AISS or 5-7 on the BSF scale will be considered as loose or watery stool.

Participants using the study product will be treated for their (infectious) disease according to common guidelines from the hospital. No adjustments in initial treatment will be made if subjects decide to participate in this study, so participating in this study will not negatively influence the initial treatment for their (infectious) disease. Besides the use of the study product, no further (invasive) interventions are needed. No extra visits to the hospital are necessary for participants. The study can be performed at home and collected samples (see next 2 paragraphs) and the diary can be picked up by one of the investigators.

In cases of the occurrence of diarrhoea during the study period, stool samples will be obtained and examined for presence of common diarrhoeal pathogens—rotavirus, adenovirus, norovirus, *Campylobacter* spp*, Salmonella* spp*, Shigella* spp*, and Yersinia* spp (—via chromatographic immunoassay for viruses or isolation from stool culture for bacteria). Additionally, *C. difficile* toxins A and B will be identified in the stool using immunoassay in cases involving children older than 1 year. Stool samples will be collected in sterile stool containers (Stuhlgefäß 10 mL, Frickenhausen, Germany).

Additionally, stool samples will be collected at home in the freezer at -20C from each participant at six time points: (1) at baseline preferably before the start of antibiotics, (2) at the day of antibiotic cessation, (3) at the end of intervention and (4) 1 month, (5) 6 months and (6) one year after the cessation of the intervention period. The collected samples will be used to conduct microbiota analysis and metabolomics. Besides this, demographic data, in particular participants' weight and length will be collected at the same time as the last stool sample and compared with data collected at baseline in both groups since microbiota dysbiosis increases the risk on obesity (45).

The observation period for primary and secondary outcomes will be identical to the intervention period and the following 7 days.

All samples from participants in Dutch centres will be transported to the Paediatric ward of the Amsterdam UMC, location VUmc and will be stored in freezers at a temperature of -20ºC. A data and material transfer agreement is made between providing centres (AMC and OLVG Oost and West) and receiver (VUmc).

In the event that a participant does not respond well to the antibiotics prescribed by the physician this can roughly be because of following reasons; (1) the micro-organism causing the infectious disease is not of bacterial origin (for example a viral infection like an otitis) or (2) there is no response to the antibiotics because the causing bacteria is resistant to the antibiotic prescribed or because of inhibition of effect of the antibiotics as a result of interaction with the probiotics.
For this last reason, if a participant does not respond well to the prescribed antibiotics and does become more ill and consults the treating physician for this reason, it will be decided whether participation should stop. If a participant develops a severe or generalised infection, symptoms of sepsis, or a critical or life-threatening illness (and thus not meeting the inclusion-criteria) the intervention will be immediately stopped and the participant will be excluded. The treating physician will inform the researcher about the termination of this individual in the study participation. If relevant for the regular treatment, the treating physician can ask for the code to be broken so it becomes evident whether the patient was using probiotics or placebo.

The timeline of this study is presented in table 1 below.

Observation and adherence to protocol recommendations

Participants will be monitored during the intervention period and for the next seven days. In cases of inpatients discharged from the hospital before the end of the intervention as well as in outpatients, the remaining product along with the study diary and the collected samples will be collected from the participant's home by one of the investigators. Compliance with the study protocol will be assessed by direct interview with the patient and/or caregiver, by analysing information from the study diary and by checking the number of returned non-consumed study products. Participants who receive <75% of the recommended dose of MSP/placebo will be considered as non-compliant and will be excluded in the further analysis.

11.4 Withdrawal of individual subjects

Subjects can leave the study at any time for any reason if they wish to do so without any consequences, and without an obligation to give reasons for the decision. The

investigator can decide to withdraw a subject from the study for urgent medical reasons.

In case of occurrence of serious adverse events or new circumstances affecting the safety of the participants (e.g. difficulty in swallowing, new diagnosis of immunodeficiency), the intervention will be discontinued.

Table 1. The timeline of the study

	**Intervention period**															
	Days of antibiotic treatment							Days after antibiotic treatment								
	1	2	3	4	5	Etc.	n (end of antibiotic treatment)	n + 1	n + 2	n + 3	n + 4	n + 5	n + 6	n + 7	n + 37 (1 month after n+7)	Follow up n + 6 months and n + 1 year
**Enrolment**																
Eligibility assessment	**x**															
Informed consent reception	**x**															
Allocation and randomisation	**x**															
Handing over study diary	**x**															
**Interventions**																
Antibiotic treatment	◄						►									
Multispecies probiotic	◄													►		
Placebo	◄													►		
**Data collection**																
Study diary	◄													►		
Stool tests in case of diarrhoea	◄													►		
Stool microbiota examination	**x**						**x**							**x**	**x**	**x**
Reception of study diary and unused product														**x**		

11.5 Specific criteria for withdrawal

The treating physician can deviate from study protocol for medical reasons, but study follow-up will continue as part of the intention-to-treat analysis. Thus no specific criteria for study withdrawal exist.

11.6 Replacement of individual subjects after withdrawal

Individual subjects will not be replaced after withdrawal. In the sample size calculation an assumed 20% withdrawal rate was taken in account.

11.7 Follow-up of subjects withdrawn from treatment

An intention-to-treat model will be applied—data from all randomised participants will be used in the analysis, including those with low compliance or those who drop out or withdraw their consent. This method analyses the occurrence of outcomes in groups to which subjects were initially assigned by randomization, regardless of whether they ultimately underwent the planned intervention or not. This method preserves the essence of randomization, i.e. the initial balance of known and unknown prognostic factors between groups. Samples obtained from participants before withdrawal will be used for the analyses too.
Per-protocol analysis will be performed as well, and it will include all participants who finish the study according to the protocol.

11.8 Premature termination of the study

We do not expect the study to be terminate prematurely. The study can be stopped prematurely if completing the study is no longer feasible. The steering committee, the Data Safety Monitoring Board, will be systematically involved in every important decision concerning conduct of the study, including discontinuation. The METC will be informed without delay if any investigator has ethical concerns about continuation of the trial.

Possible criteria for terminating the study prematurely:

- Early evidence that the investigational product is beneficial for the conditions studied or solid statistical evidence that an the investigational product is better than the comparator
- Early evidence that the investigational product is, in contrary, harmful (ADRs, SAEs, SUSARs, etc.)
- If an interim statistical analysis showed that a clinical trial has no scientific (statistical) value/power
- It is not feasible to reach the planned outcomes.
- Loss of extramural funding

- difficulty in completing the study as originally planned
- Other reasons pertaining to the efficacy, safety, or feasibility of the study

12 SAFETY REPORTING

Monitoring

The study will be carried out in accordance with the protocol, as it will be registered. No changes in the study protocol are expected to be made after the study starts. However, in case of any unexpected circumstances requiring alterations of the protocol, changes will be immediately applied to the protocol registry site at clinicaltrials.gov, and, if relevant enough, reported to the Bioethics committee. An independent Data and Safety Monitoring Board (DSMB) will be created before the start of the study. The DSMB will review data every half year to assess the study progress (including rate of recruitment, completeness of data and their appropriate collection) and all of the SAE's. The number of recruited patients will be monitored and kept up to date (see section 12.5); appropriate changes (i.e., training of the recruiting physicians, study leaflets, addition of new recruitment centres) will be applied to the study procedure and protocol if the pace of recruitment is not high enough to finish the study within the established time, which is 2 years.

Harms

All eight of the probiotic strains to be used in the study have the Qualified Presumption of Safety status (46) established by the European Food Safety Authority (EFSA). The occurrence of serious adverse events in immunocompetent populations during oral use of probiotics is unlikely (47).

The exact same product has not been assessed in previous studies. However, several clinical studies have been performed with a comparable product, in different populations (healthy volunteers and chronic obstructive pulmonary disease patients) in the Netherlands and Austria without any reported serious side effects (18-21, 28). In addition, the preparation is commercially available in several countries (Austria, Germany, Greece, Norway, Russia, Slovenia, Ukraine and the Netherlands) and since the market introduction in 2007, no serious adverse effects have been reported.

In the Netherlands, probiotics are considered to be food or food supplements and, therefore, have to be produced under Hazard Analysis and Critical Control Point regulations, which is the Dutch regulation system for safety and hygiene in food and food supplements. Winclove is an NSF International Certified Good Manufacturing Practices Facility for manufacturing dietary supplements and works with the food safety management system ISO 22000:2005.
In case of suspected serious adverse events, the project leader will immediately notify the Ethics Committee, DSMB, all study personnel and the manufacturer of the product about the nature of the event. The decision regarding continuation or

discontinuation of the trial will be made by the project leader in agreement with the Ethics Committee and DSMB.

12.1 Temporary halt for reasons of subject safety

In accordance to section 10, subsection 4, of the WMO, the sponsor will suspend the study if there is sufficient ground that continuation of the study will jeopardise subject health or safety. The sponsor will notify the accredited METC without undue delay of a temporary halt including the reason for such an action. The study will be suspended pending a further positive decision by the accredited METC. The investigator will take care that all subjects are kept informed.

12.2 AEs, SAEs and SUSARs

12.2.1 Adverse events (AEs)

Adverse events are defined as any undesirable experience occurring to a subject during the study, in any way being considered related to the investigational product. All adverse events reported spontaneously by the subject or observed by the investigator or his staff will be recorded. Adverse events will be recorded until one month after the termination of the use of the investigational product.

12.2.2 Serious adverse events (SAEs)

A serious adverse event is any untoward medical occurrence or effect that

1. results in death;
2. is life threatening (at the time of the event);
3. requires hospitalisation or prolongation of existing inpatients' hospitalisation;
4. results in persistent or significant disability or incapacity;
5. is a congenital anomaly or birth defect; or
6. any other important medical event that did not result in any of the outcomes listed above due to medical or surgical intervention but could have been based upon appropriate judgement by the investigator.

An elective hospital admission will not be considered as a serious adverse event.

The investigator will report all SAEs to the sponsor without undue delay after obtaining knowledge of the events.

The sponsor will report the SAEs through the web portal *ToetsingOnline* to the accredited METc that approved the protocol, within 7 days of first knowledge for SAEs that result in death or are life threatening followed by a period of maximum of 8 days to complete the initial preliminary report. All other SAEs will be reported

within a period of maximum 15 days after the sponsor has first knowledge of the serious adverse events.
Serious adverse events will be recorded until one month after the termination of the use of the investigational product.

12.2.3 Suspected unexpected serious adverse reactions (SUSARs)

Not applicable as we will not investigate a medicinal product. Winclove 612 is considered to be a food supplement/product.

12.3 Annual safety report

Not applicable.

12.4 Follow-up of adverse events

All AEs will be followed until they have abated, or until a stable situation has been reached. Depending on the event, follow up may require additional tests or medical procedures as indicated, and/or referral to the general physician or a medical specialist. SAEs will be reported till end of study within the Netherlands, as defined in the protocol.

12.5 Data Safety Monitoring Board (DSMB)

An independent Data and Safety Monitoring Board (DSMB) will be created before the start of the study. The DSMB will be established to perform ongoing safety surveillance and to perform interim analyses on the safety data. This committee will be an independent committee. The members have no conflict of interest with the sponsor of the study.
The project leaders had contacted the Clinical Research Bureau (CRB) from the Amsterdam UMC, location VUmc about the formation of the DSMB. Usually a DSMB is only facilitated by the CRB when the study is a classified as a ‘high-risk study’. This study is not classified as a ‘high-risk’ study. However, since it involves children it was decided, in consultation with the CRB, that the CRB will facilitate a DSMB for this study.

The DSMB will review data after recruitment every half year to assess the study progress (including rate of recruitment, completeness of data and their appropriate collection) and all of the adverse events.

A separate Charter will describe the roles and responsibilities of the independent DSMB, including the timing of meetings, methods of providing information to and from the DSMB, frequency and format of meetings and relationships with other committees.

The advice(s) of the DSMB will only be sent to the sponsor of the study. Should the sponsor decide not to fully implement the advice of the DSMB, the sponsor will send the advice to the reviewing METC, including a note to substantiate why (part of) the advice of the DSMB will not be followed.

The DSMB will consist of:

1. A chair of the DSMB:
   - Prof. dr. P. Huijgens
2. An independent researcher with sufficient knowledge on statistics and epidemiology:
   - Prof. dr. Boers
3. An independent doctor with sufficient clinical knowledge on the topic of this study.
   - Dr. M. van der Kuip

The members of the DSMB will be formed by the CRB. The CRB has confirmed that they will provide members as described above.

No DSMB member has any conflict of interest with Winclove Probiotics B.V.

13 STATISTICAL ANALYSIS

Subject baseline and demographic data, as well as baseline disease characteristics data will be summarized for all subjects by treatment regimen and compared between the two groups.

For continuous variables, comparison between groups will be done using the Student's t-test or Mann-Whitney U test, depending on whether or not the variables are distributed normally. The χ^2 test or Fisher's exact test will be used, as appropriate, to compare dichotomous variables. Differences between groups will be presented for continuous outcomes as differences in means or differences in medians (for normal or non-normal distribution, respectively) along with a 95% CI. For dichotomous outcomes, the relative risk (RR) and number needed to treat, calculated as the inverse of the absolute risk reduction, will be determined along with a 95% CI. The difference between study groups will be considered significant when the p value is <0.05.

An intention-to-treat model will be applied—data from all randomised participants will be used in the analysis, including those with low compliance or those who drop out or withdraw their consent. This method analyses the occurrence of outcomes in groups to which subjects were initially assigned by randomization, regardless of whether they ultimately underwent the planned intervention or not. This method

preserves the essence of randomization, i.e. the initial balance of known and unknown prognostic factors between groups. Per-protocol analysis will be performed as well, and it will include all participants who finish the study according to the protocol.

13.1 Primary study parameter(s)

Primary outcome

The incidence of AAD, defined as three or more loose or watery stool per day in a 24-hour period, either caused by *C. difficile* infection or of otherwise unexplained aetiology	χ^2 test or Fisher's exact test with relative risk and number needed to treat, along with a 95% CI.

13.2 Secondary study parameter(s)

Variable distribution will be assessed by Shapiro-Wilk tests. Depending on the results of these tests, data will be analyzed by either parametric or non-parametric statistics.

The incidence of AAD, defined as three or more loose or watery stools per day (defined as above) for a minimum of 48-hour period either caused by *C. difficile* infection or of otherwise unexplained aetiology.	χ^2 test or Fisher's exact test with relative risk and number needed to treat, along with a 95% CI.
The incidence of AAD, defined as two or more loose or watery stools per day (defined as above) for a minimum of a 24-hour period either caused by *C. difficile* infection or of otherwise unexplained aetiology.	χ^2 test or Fisher's exact test with relative risk and number needed to treat, along with a 95% CI.
The duration of diarrhoea (in days)	Student's t-test or Mann-Whitney U test with differences in means or differences in medians along with a 95% CI.
Incidence of any diarrhoea, regardless of its aetiology	χ^2 test or Fisher's exact test with relative risk and number needed to treat, along with a 95% CI.
Incidence of *C. difficile*-associated diarrhoea	χ^2 test or Fisher's exact test with relative risk and number needed to treat, along with a 95% CI.

Discontinuation of the antibiotic treatment due to severity of diarrhoea	χ^2 test or Fisher's exact test with relative risk and number needed to treat, along with a 95% CI.
Hospitalisation caused by diarrhoea in outpatients	χ^2 test or Fisher's exact test with relative risk and number needed to treat, along with a 95% CI.
Need for intravenous rehydration	χ^2 test or Fisher's exact test with relative risk and number needed to treat, along with a 95% CI.
Adverse events	χ^2 test or Fisher's exact test with relative risk and number needed to treat, along with a 95% CI.
Intestinal microbiota composition<br><br>Amino acid analysis<br><br>Metabolomics analysis	The species diversity (ɑ-diversity) of faecal samples will be calculated using Faith's phylogenetic diversity. The Shannon-diversity indices, absolute and relative abundances, unsupervised and supervised classification methods for diversity applying correction for the differences in sequencing depths by rarefaction will be used.

13.3 Other study parameters

Not applicable

13.4 Interim analysis

The DSMB will review data every half year as decribed in the additional DSMB charter.

14. ETHICAL CONSIDERATIONS

Ethics and dissemination

The protocol of the study will be reviewed by the Ethics Committee (METc) of the Amsterdam UMC, location VUmc. Participants (or their legal representatives) will be fully informed about the study, and informed consent will be obtained. The full protocol is published in a peer-reviewed journal and available online (22). The results of the study will also be published in a peer-reviewed journal and submitted for presentation on conferences related to the topic of the thesis. The results will be reported in accordance with the Consolidated Standards of Reporting Trials (CONSORT).

14.1 Regulation statement

This study will be conducted according to the principles of the Declaration of Helsinki (WMA Declaration of Helsinki – Ethical Principles for Medical Research Involving Human Subjects Adopted by the 18th WMA General Assembly, Helsinki, Finland, June 1964 and amended by the 64th WMA General Assembly, Fortaleza, Brazil, October 2013)) and in accordance with the Medical Research Involving Human Subjects Act (WMO).

14.2 Recruitment and consent

The supervising physician will inform researchers whether a patient is eligible to participate or not. In the event a patient seems eligible, the treating physician will contact one of the researchers. He or she will then further inform participants. Participants will both orally by the researcher and in writing via a patient information letter (PIL), be informed about the study. In the information letter participants and/or parent(s) and/or caregiver(s) are asked for informed consent. Participants will have 24 hours after taking the antibiotics to consider their decision in participating. It will be clearly stated that the participant is free to withdraw from the study at any time for any reason without prejudice to future care, and with no obligation to give the reason for withdrawal. Participant Information Sheets will be available in Dutch. Written Informed Consent will be confirmed by the dated signatures of the participant and by the person who presented and obtained the informed consent. The person obtaining consent must be suitably qualified and experienced, and be authorized to do so by the Principal Investigator. A copy of the signed Informed Consent will be given to the participants. The original signed form will be retained at the study site.The patient information letter and informed consent form will be attached as a separate document.
'Participants will also be recruited via pharmacies connected to the FBA (Farmaceutisch bureau Amsterdam) region Amsterdam-Amstelland. The same patient population with the same in- and exclusion criteria will be approached and included via this route, to increase the inclusion rate. The pharmacist who hands out antibiotics, prescribed by paediatricians and general practitioners, will also hand out a flyer with information about this study (see document E3) and with contact information of the researchers. After handing out the flyer by the pharmacist, all study procedures will be done exactly the same as currently done for patients recruited via the treating physician. If patient contact the researchers via the flyer with interest to participate in this study, the investigators will check whether a patient is eligible to participate (the investigator will check the in- and exclusion criteria) and provide informed consent. Participants will be informed about the study and instructed by the researcher (by telephone) and in writing via a patient information letter (PIL). Consequently, potential inclusions will receive exactly the same information as participants that are being recruited by the treating physician in

the Amsterdam UMC. In the information letter participants and/or parent(s) and/or caregiver(s) are asked for informed consent. Participants will have 24 hours after initiation of the antibiotics to consider their decision in participating. It will be clearly stated that the participant is free to withdraw from the study at any time for any reason without prejudice to future care, and with no obligation to give reason for withdrawal. Participant Information Sheets will be available in Dutch. Written Informed Consent will be confirmed by the dated signatures of the participant and by the person who presented and obtained the informed consent. The person obtaining consent must be suitably qualified and experienced, and be authorized to do so by the Principal Investigator. A copy of the signed Informed Consent will be given to the participants. The original signed form will be retained at the study site.The patient information letter and informed consent form will be attached as a separate document.
When participants are recruited via the pharmacist, the investigator will report the inclusion to the treating physician.

For this study there are strict exclusion criteria about starting the study. If a patient has been using antibiotics for >24 hours before both parents have given written informed consent a patient needs to be excluded. After an inclusion period of 9 months, a lot of children had to be excluded because both parents did not give written consent in time, even though both of them gave oral consent for participation.
To overcome this problem, we want to already hand out the study products before both parents/caregviers have given written informed consent in one exceptional situation. In the case when only one parent/caregiver is present with the child and able to sign the informed consent form directly and the other parent is unable to sign directly or within 24 hours after the first dose of antibiotics is taken by the child (1st dose of antibiotic used >24 hours ago is one of the exclusion criteria), the first parent can already sign the informed consent form and declare that the second parent/caregiver has given oral consent for participation of the child. The investigator will make sure that the second parent is aware of the study and the study protocol, and also the second parent was given the possibility to ask his/her questions by phone. The second parent will be informed orally by the researcher via the telephone and in writing via a digital patient information letter (PIL) about the study. Only in this case, when one of the parents/caregivers has given written informed consent, and the second parent/caregiver gives oral consent for participation, the investigator may already hand out the study products and the investigation can start before both parents have given written informed consent.

14.3 Objection by minors or incapacitated subjects

It is possible that a child will resist to participate. Section 4, subsection 2, of the WMO stipulates that a minor or legally incompetent adult cannot be forced to undergo a treatment or behave in a particular manner in the context of non-therapeutic research against his or her will. In the patient information letter the grounds on which a subject will be deemed to object are stated. In case of resistance to participating from a minor, the researcher will stop the study in this particular subject. We will adhere to The Netherlands Association for Pediatric Medicine (NVK) 'Code of conduct relating to expressions of objection by minors participating in medical research' which can be found on the website of the CCMO and to the European Medicines Agency guidelines for good clinical practice.

14.4 Benefits and risks assessment, group relatedness

Based on all clinical trials performed with comparable products, patients can expect that by using the investigational product less diarrhoea-like bowel movements will occur, and microbiota is restored or will even prevent dysbiosis, consequently preventing AAD. The potential risks of participation are low, especially as probiotic intervention is started after critical or life-threatening illness are excluded. No serious events are expected from administration of the test product, or control product. If adverse effects occur, they are likely to be minor gastro-intestinal symptoms, such as bloating or flatulence, especially when one uses probiotics for the first time. These effects are expected to disappear after a few days.

As mentioned earlier all substances of the used products are considered safe for use in children. There have been several studies with comparable products which do not report any serious side effects. Besides this, a quite comparable product to the investigational product is available in many countries, and on the Dutch market since 2007 without any serious side effects being reported.

AAD is a common complication of antibiotic treatment in children. Certain probiotic strains are proven to be effective in reducing risk of AAD, however, there is not enough evidence to recommend use of a MSP. The benefit-risk ratio of this MSP needs to be determined in children. With this study we want to contribute to better insight in the effectiveness of MSP's in the prevention of AAD this population. As response to probiotic treatment might differ between children and adults, this study cannot be performed in adult patients. Since this study will lead to important knowledge on preventing AAD in children (and the risks and burden of participating are low), we think that the risk to and burden for the subject will be in proportion to the potential value of the research.

14.5 Compensation for injury

Insurance for the participants

Participants are insured against any potential damages incurred as a result of participating in the study. Amsterdam UMC, location VUmc covers the WMO subject insurance by a continuous coverage.

The investigator has a liability insurance which is in accordance with article 7 of the WMO.

The sponsor (also) has an insurance which is in accordance with the legal requirements in the Netherlands (Article 7 WMO). This insurance provides cover for damage to research subjects through injury or death caused by the study.

1. € 650.000,-- (i.e. four hundred and fifty thousand Euro) for death or injury for each subject who participates in the Research;
2. € 5.000.000,-- (i.e. three million five hundred thousand Euro) for death or injury for all subjects who participate in the Research;
3. € 7.500.000,-- (i.e. five million Euro) for the total damage incurred by the organisation for all damage disclosed by scientific research for the Sponsor as 'verrichter' in the meaning of said Act in each year of insurance coverage.

The insurance applies to the damage that becomes apparent during the study or within 4 years after the end of the study.

14.6 Incentives

Participants will receive no financial compensation for participating. However, participants (children) will receive a small compensation/gift for their participation. Participants will receive either one free entrance ticket for the NEMO Science Museum Amsterdam or a small teddy bear from IKEA (participants and/or parents are allowed to choose one of two available gifts). No extra visits are warranted, so there won't be any travel or parking costs for participants. Remaining study products and collected samples can be transported to the study site at a regular appointment or can be picked up at home by one of the investigators.

15 ADMINISTRATIVE ASPECTS, MONITORING AND PUBLICATION

15.1 Handling and storage of data and documents

Data will be handled confidentially. The handling of patient material and data will comply the EU General Data Protection Regulation and the Dutch Act on Implementation of the General Data Protection Regulation. (in Dutch: Uitvoeringswet AVG, UAVG).). All study data and human material will be handled confidentially and coded with a unique study number to protect the subject privacy. Data will be coded on sequence of inclusions per participating centre, e.g. AMC001, AMC002, OLVG001, OLVG002 et cetera. A subject identification code list will be used to link the data to the subject. Only the research team (i.e. principal investigators, research nurse,

researcher and those otherwise involved in this study) will be able to identify the participants and will have access to the data and human material. The key to the code will be safeguarded by the investigator. Data will be recorded on a secure password-protected electronic case record form (eCRF) only on protected servers in the Amsterdam UMC, location VUmc, which will be managed and checked by the researcher. These data will be transferred to a computer system for subsequent tabulation and statistical analysis. Data and human material will be coded and stored during the study period. When patients and their parents/caregivers give permission, this data will be stored for a period of 15 years. Only the principal investigator and other investigators involved in the study will have access to the samples. These samples will be used to answer current research questions as well as possible new research questions that fall within the scope of this study. For future research that falls outside the scope of this study, participants will be asked new permission to use these samples.
Stool samples from the 175 participants in the Netherlands can be collected at home or in the hospital when a patient is admitted to the ward. Samples will be transported to the Amsterdam UMC, location VUmc and will be coded. A subject identification code list will be used to link the data to the subject. Only the research team (i.e. principal investigators, research nurse, researcher and those otherwise involved in this study) will be able to identify the participants and will have access to the data and human material. The key to the code will be safeguarded by the investigator. Samples will be stored in to a special biobank of the Amsterdam UMC, location VUmc. Samples will be stored here for a maximum of 15 years after written informed consent is obtained to do so.

15.2 Monitoring and Quality Assurance

The DSMB will review data after recruitment from 25%, 50% and 75% of participants to assess the study progress (including rate of recruitment, completeness of data and their appropriate collection) and all of the adverse events. The number of recruited patients will be monitored and kept up to date.
We refer to section 12.5 to the exact tasks of the DSMB and what will be included in the interim analysis.

Monitoring of the coordinated investigator will be done by a GCP-certified research nurse, PhD candidate and/or the DSMB. The monitor will provide a written report to the coordinated investigator after each visit including a summary of the significant findings, deviations and deficiencies, conclusions, actions taken or recommended to secure compliance. The coordinated investigator will run consistency checks on a monthly basis and produce queries to be resolved by the local investigator(s). The final database will be obtained after the resolution of all queries.

15.3 Amendments

Amendments are changes made to the research after a favourable opinion by the accredited METC has been given. All amendments will be notified to the METC that gave a favourable opinion.

15.4 Annual progress report

The investigator will submit a summary of the progress of the trial to the accredited METC once a year. Information will be provided on the date of inclusion of the first subject, numbers of subjects included and numbers of subjects that have completed the trial, serious adverse events/serious adverse reactions, other problems, and amendments.

15.5 Temporary halt and (prematurely) end of study report

The investigator will notify the accredited METC of the end of the study within a period of 8 weeks. The end of the study is defined as the last patient's last visit.

The sponsor will notify the METC immediately of a temporary halt of the study, including the reason of such an action.

In case the study is ended prematurely, the sponsor will notify the accredited METC within 15 days, including the reasons for the premature termination.

Within one year after the end of the study, the investigator will submit a final study report with the results of the study, including any publications/abstracts of the study, to the accredited METC.

15.6 Public disclosure and publication policy

Results of this study will be presented on national and international conferences and in national and international medical journals, within the scope of the target group, namely (pediatric) gastroenterologists. The sponsor of this study will not have any influence on data analysis and on publication of results. The authors have no conflict of interest. The project will be registered at clinicaltrials.gov (NCT03334604).
The Investigators will be involved in reviewing drafts of the manuscripts, abstracts, press releases and any other publications arising from the study. Authorship will be determined in accordance with the ICMJE guidelines and other contributors will be acknowledged. The trial will be registered at clinicaltrials.gov before the first patients is recruited. Research will be conducted in accordance with the AMC-VUmc research code.

16 STRUCTURED RISK ANALYSIS

16.1 Potential issues of concern

Based on all clinical trials performed (18-21, 28, 29), patients can expect that by using Winclove 612 less diarrhoea-like bowel movements might occur, microbiota might be restored or dysbiosis will even be prevented.

The potential risks of participating are expected to be low, especially as probiotic intervention is started after bacteraemia and sepsis are excluded. No serious events are expected from administration of the test product or control product. However, this can never be completely be ruled out. If adverse effects occur, they are likely to related to minor gastro-intestinal symptoms, such as bloating or flatulence, especially when one uses probiotics for the first time. These effects are expected to disappear after a few days. If more serious adverse events occur, or if patients become more ill than at the start of the study the treating physician can decide that a patients need to be excluded from further participation.

a. Level of knowledge about mechanism of action

The pathogenesis of AAD is not fully understood. It may be caused by a specific enteric pathogen (e.g., *Clostridium difficile, Clostridium perfringens, Staphylococcus aureus, Candida albicans*), metabolic consequences of altered intestinal microbiota or a direct effect of antibiotics on the mucosa (9). The impact of antibiotics on the abundance of commensal micro-organisms in the gut underlines the hypothesis that administration of probiotics could reduce the incidence of AAD. Probiotics are 'live microorganisms that provide health benefits to the host when ingested in adequate amounts' (11). Probiotics are demonstrated to be used as therapeutic options for a variety of diseases, but the mechanisms responsible for these effects have not been fully elucidated yet. Several important mechanisms underlying the antagonistic effects of probiotics on various microorganisms include the following: modification of the gut microbiota, competitive adherence to the mucosa and epithelium, strengthening of the gut epithelial barrier and modulation of the immune system to convey an advantage to the host. Accumulating evidence demonstrates that probiotics communicate with the host by pattern recognition receptors, such as toll-like receptors and nucleotide-binding oligomerization domain-containing protein-like receptors, which modulate key signaling pathways, such as nuclear factor-κB and mitogen-activated protein kinase, to enhance or suppress activation and influence downstream pathways. This recognition is crucial for eliciting measured antimicrobial responses with minimal inflammatory tissue damage (37, 48). It is plausible that by using probiotics during antibiotic treatment less diarrhoea-like bowel movements will occur, and microbiota is restored or even prevent dysbiosis, consequently preventing AAD.

<u>b. Previous exposure of human beings with the test product(s) and/or products with a similar biological mechanism</u>

Ecologic AAD has been available at drugstores and pharmacies in many countries including the Netherlands since 2007. Since the introduction on the market, no serious side effects have been reported.
A double-blind, placebo controlled randomized controlled trial showed that Ecologic AAD is able to significantly reduce the risk of diarrhoea-like defectation (18). Bowel movements and/or a loose consistency were reported less frequently. Changes over time were present in both groups regarding microbial composition and metabolic activity, which differed significantly between the probiotic and the placebo arm, suggesting that the effect of antibiotics was modulated by probiotic intake.
In another RCT with 30 COPD patients the investigated MSP did not affect the composition of the dominant faecal microbiota nor the occurrence of diarrhea-like bowel movements. The dominant faecal microbiota was not affected by antibiotics in this COPD population, suggesting an existing imbalance of the microbiota, which may also have contributed to the lack of effect by probiotic intake (19).
In a study with 199 patient receiving antibiotics and a MSP, only 2 patients developed AAD which probably had been caused by overgrowth of *Clostridium difficile* in one of the patients. Based on the knowledge from literature the incidence of AAD ranges from 5-39% in the population used in that study. An observed almost complete absence of AAD incidence (0.5%) in this cohort of Ecologic AAD treated patients strongly suggests a beneficial role of Ecologic AAD therapy during and after antibiotic treatment (20).
In a retrospective case report series of 10 patients with *Clostridium difficile* infection, 5 of whom experienced recurrent CDI, all patients received, besides antibiotics Ecologic AAD, which resulted in complete recovery. Moreover no adverse events were reported (21).
In their global guidelines, the World Gastroenterology Organisation (WGO) evaluates pro- and prebiotics on their efficacy and safety. In this guideline Ecologic AAD is evaluated as *a randomized trial with dramatic effect in the prevention of antibiotic-associated diarrhea (27)*. More specifically, the evidence for Winclove's probiotic formulation Ecologic AAD is evaluated as level 2 evidence: Randomized trial or observational study with dramatic effect. The recommendation of Ecologic AAD as an effective and safe probiotic for reducing antibiotic-associated diarrhea by the WGO is very promising in implementing probiotics as a standard prescription next to antibiotics.
For a more detailed description of the studies on the efficacy and safety of we refer to chapter 9.3.

<u>c. Can the primary or secondary mechanism be induced in animals and/or in *ex-vivo* human cell material?</u>

The pathogenesis of AAD is not fully understood, and unfortunately, no good animal model has been found until now. However there are some studies investigating the effect of probiotics *in vitro*. It can be concluded that most strains in Winclove 612 are very capable to inhibit one or more pathogens likely to cause AAD.
However, there are also some studies with opposite conclusions. In one study with mice. spontaneous post-antibiotic recovery compared to probiotics, the latter induced a markedly delayed and persistently incomplete indigenous stool/mucosal microbiome reconstitution and host transcriptome recovery toward homeostatic configuration, while autologous fecal microbiome transplantation induced a rapid and near-complete recovery within days of administration. In this study, *in vitro*, *Lactobacillus*-secreted soluble factors contributed to probiotics-induced microbiome inhibition. These contradictory results show the need of good quality clinical studies involving humans and are a rationale to perform the proposed study.
For a more detailed description of the studies investigating the effect of the strains *in vitro* we refer to chapter 9.2.

d. Selectivity of the mechanism to target tissue in animals and/or human beings
Probiotics will be present in different concentrations at different levels of the gastro-intestinal tract (see paragraph 16.2 f). They will not be absorbed nor target other tissues and thus will only have an effect on tissues in the gastro-intestinal tract, specifically in the intestines. The probiotics can also be present in a different amount of CFU in the stool of the participants. This will be further discussed in 16.2 Synthesis.

e. Analysis of potential effect
The direct effect of probiotics is on tissues in the gastro-intestinal tract, mostly in the gut. Here, probiotics may exert its beneficial effects by enhancing gut barrier function, inhibiting colonization of potentially pathogenic microorganisms, maintaining a normal intestinal milieu, synthesizing antibacterial substances and stimulating local immunity.
Based on all clinical trials performed with Ecologic AAD as very comparable product to the current investigational product Winclove 612 and the studies mentioned in 16.1b, patients can expect that by using Winclove 612 less diarrhoea-like bowel movements will occur, and microbiota might restored or even prevent dysbiosis, consequently preventing AAD.
The potential risks of participation are expected to be low, especially as probiotic intervention is started after critically ill patients are excluded. No serious events are expected from administration of the test product, or control product. If adverse effects occur, they are likely to related to minor gastro-intestinal symptoms, such as bloating or flatulence, especially when one uses probiotics for the first time. These effects are expected to disappear after a few days (18-21, 28). So the adverse effects are expected to be mild and the benefit of treatment seems to outweigh this

risk. As mentioned in paragraph 9.3 and 16.1b, it is thought that probiotics have a beneficial effect on the microbiome, however there are also studies claiming otherwise and that more data is needed to elucidate this. With this study we want to contribute to evidence.

f. Pharmacokinetic considerations

The product has a concentration of $2.5*10^9$ CFU/gram with an advised daily dosage of 2 grams twice daily. The dosage of Winclove 612 is in accordance with most probiotic dosages used in human studies and many commercially available products, ranging from 10^9-10^{10} CFU/dose and by the recommendations of the TNO (29). It is not possible to state a general dose for probiotics; some have shown to be effective at lower levels, while other require substantially more. The dosage of Winclove 612 is this study is based on mentioned studies and recommendations and on prior human studies in healthy volunteers, COPD patients and general surgery patients with the product (18-21, 28) showing a health benefit without adverse reactions.
Probiotic strains differ in their survival capacity at different levels of the gastrointestinal tract (37). The concentration of probiotics needed to obtain a clinical effect is often quoted as $\geq 10^6$ colony-forming units/ml (CFU) in the small bowel and $\geq 10^8$ CFU/g in the colon (36). Dose–response studies are, however, scarce, and it is not known whether the percentage survival is stable for various ingested doses.
The survival of probiotics depends on their intrinsic resistance, on host factors and on the vehicle in which they are ingested. The main obstacles to survival being gastric acidity and the action of bile salts. Well-controlled, small-scale studies on diarrhea in both adults and infants have shown that probiotics survive in sufficient numbers to affect gut microbial metabolism (37). Survival rates have been estimated at 20–40% for selected strains, but differ between strains (38). Approximately 1–10% of *Lactobacillus acidophilus* ingested in fermented products was found to survive until the ileum in several human studies using intestinal intubation techniques (39). In contrast, 30% of ingested *Bifidobacterium* was found in the colon (40). Other studies in healthy volunteers with different probiotic preparations showed that the faecal concentrations of ingested *Lb. acidophilus*, *Lactobacillus salivarius, Lactobacillus rhamnosus* strain GG reached around 10^6 CFU/g. Probiotics are usually excreted within a few days of their ingestion in faeces at the same rate as or even more quickly than a transit marker (37). Based on the best evidence, a strictly selected combination of strains is used in this study.

g. Study population

The study population will consist of healthy children aged between 3 months and 18 years old (see inclusion and exclusion criteria). Participants will be recruited among both the inpatient and outpatient clinics from the department of Paediatrics in the participating centres in Amsterdam. The condition of the patients that participate in the study is stable.

Since our study aim is to investigate the effect of probiotics on AAD in children, we will include only children in this study. Response to probiotic treatment might differ between children and adults, so this study cannot be performed in adult patients.

h. Interaction with other products
Participants will not be given a combination of products. There is no evidence suggesting that oral administration of the used probiotics may alter the beneficial effect of antibiotics or vice versa.

i. Predictability of effect
Predictive biomarkers for the effect and adverse effect of probiotics are not available.

j. Can effects be managed?
Ecologic AAD is available in drugstores in a lot of countries including The Netherlands since 2007. Possible expected side effects from Ecologic AAD and Winclove 612 are mild gastro-intestinal symptoms. These symptoms will disappear in a few days. Physicians are experienced in coping with symptoms similar to the possible side effects of probiotics.

16.2 Synthesis

In summary the overall risk of participation in this trial is thought to be low. Probiotics are generally regarded as safe. Recent safety studies have concluded that the consumption of probiotics is well-tolerated and generally recognized as safe across all age groups (49, 50). However it has to be acknowledged that not all studies on probiotics reported adverse thoroughly (32). Studies reporting side effects and negative consequences of probiotic use showed no major safety concerns, as none of the serious adverse events were related, or suspected to be related, to the probiotic or synbiotic product and the study products were well tolerated. Despite this, we took some additional safety measures mentioned later on in this synthesis to secure participants safety.

Studies on the efficacy of probiotics on the incidence of AAD are controversial: there are studies claiming that probiotics should be used to prevent dysbiosis and AAD, however some studies questioning the effectivity of probiotics. One of these studies showed that probiotics induced a markedly delayed and persistently incomplete indigenous stool/mucosal microbiome reconstitution after antibiotic use (34). Is has to be noted however that a probiotic strain with strains of the gena *Lactococcus* and *Streptococcus* were used; two strains with less evidence of their efficacy compared to *Bifidobacteria* and *Lactobacilli* strains. The hypothesis is that probiotics can be disadvantageous if used in wrong dosage or if the wrong strains are used. Therefore,

it is even more important that good-quality studies are being performed on the impact of probiotic strains on the microbiome.
A systematic review of relevant clinical studies for effective probiotics (the practical guide for the use of probiotics for the prevention of AAD by TNO) mentions that studies with *Bifidobacterium bifidum*, *Bifidobacterium lactis*, *Enterococcus faecium*, *Lactobacillus acidophilus*, *Lactobacillus paracasei*, *Lactobacillus plantarum*, *Lactobacillus rhamnosus*, *Lactobacillus salivarium* (minimal daily dose: $1x10^{10}$ billion CFU) show a trend to the reduction in the incidence of AAD (29). All these strains are present in our investigational product except for *Enterococcus faecium* due to safety concerns by the ESPGHAN. We are using the recommended dose by TNO. As mentioned previously, with current knowledge, it is impossible to say what the best dose and whether a different set of species should be used or in a different combination of CFU per species. However, all strains were strictly selected as mentioned previously and are being used in the recommended dose based on the best available evidence. With this study, we want to contribute to evidence of the efficacy of these selected probiotics on the incidence of AAD. Furthermore, the exact pathophysiology remains unclear. Since we also perform microbiota and metabolomic analysis, we will contribute to information on the mechanisms on how probiotics affect the microbiota. Besides this, we will be able to tell whether the diversity is affected by the probiotic strains and whether the microbiota of children who were given probiotics will resemble a more healthy microbiota compared to the microbiota of the placebo group. It is possible that the first group will have a more stable, and more healthy like microbiota. In a previous study, the composition, stability and diversity of healthy children in our population was investigated (51). Furthermore, they described healthy and commensal bacteria. With this information we can investigate whether probiotics contribute to having a more stable and diversity microbiota in children receiving antibiotics. Since dysbiosis is associated with numerous clinical conditions, prevention or quick restoration of dysbiosis might have long term positive consequences.

It could also be possible that the probiotic strains will be present in different concentrations or CFU in the stool of children. Some researchers suggest that person-, region and/or strain-specific mucosal patterns and that some people can be probiotic resistant. However, they also acknowledge that information and studies on responders and non-responders is scarce and controversial (52). If we do find a difference in CFU in the stool of children treated with probiotics, we want to zoom in on these children and compare if it correlates with other variables such as the incidence of AAD and the overall diversity of the microbiota. This way, we want to investigate whether there are clinical variables that can predict if a child will respond to probiotics.
All of the mentioned results could also be a rationale for future studies.

All of the 8 probiotic strains to be used in the study have the Qualified Presumption of Safety (QPS) status established by the European Food Safety Authority (EFSA)

(53), or have an extensive safety dossier. QPS is similar in concept and purpose as the Generally Recognized As Safe (GRAS) definition used in the USA, but modified to take into account the different regulatory practices in Europe. Experts regard all probiotic species with GRAS of QPS status as safe for consumption.

With probiotics, whether there is long-term replacement of indigenous microbes by other species or inherent advantages given to the existing microbiota to repopulate is still unclear(52, 54). With this study we want to gain more insight in the microbiota composition, how this is affected by antibiotics and probiotics. Furthermore, we contribute with good quality evidence on the effect of probiotics on the incidence of AAD. By collecting and analyzing the follow up samples we hope to get more insight in how the microbiota changes/restores after time and whether this is different between both groups. With this knowledge, the use of probiotics in children receiving antibiotics might be implemented in guidelines in the future.

Primary research studies and meta-analyses have suggested that probiotics decrease the duration of antibiotic-associated diarrhea, however evidence from these studies is of variable quality, and the mechanism underlying the clinical benefits remains unknown (52, 54). With this blinded randomised study, we want to contribute to good quality evidence on this topic and to get more insight whether there are specific populations would benefit most from this therapy and whether there are specific groups of patients that would be resistant to probiotics or where the restoration of the dysbiosis is even delayed. In our opinion, this study will result in valuable information an evidence whether there is a need of more studies and whether the use of probiotics should be implemented in guidelines on not.
Even though we expect the risks of participating to be low. We took some additional safety measurements to reduce the risks of participating. Our study population consist of stable children (see inclusion and exclusion criteria; no critical ill children will be included). If it becomes evident that children do not response well to the antibiotics and become more ill, the participant will be immediately withdrawn from the study. Besides these safety measures, we established a DSMB to perform ongoing safety surveillance. In the Netherlands, probiotics are considered to be food or food supplements and therefore have to be produced under Hazard Analysis and Critical Control Point (HACCP) regulations, which is the Dutch regulation system for safety and hygiene in food and food supplement. All components are legally admitted as food additives or food components. Winclove works with the food safety management system ISO22000:2005 and is certified for the development and production of pre- and probiotics. This specific multispecies probiotic mixture was chosen, of which multiple previous clinical studies have been performed showing no increase in adverse events.

Possible side effects expected from the use of Winclove 612 are expected to be mild gastro intestinal symptoms such as bloating or flatulence which are expected to

disappear after a few days. Besides this, children participating may even benefit by participating as AAD may be prevented by using probiotics.

Since this study will lead to an important gain of knowledge on preventing AAD in children and on the effects of antibiotics and probiotics on the microbiota and the risks and burden of participating are expected to be low, we deem the risk to and burden for the subject is in proportion to the potential benefit and value of the research. The knowledge obtained about the effects of probiotics on AAD could potentially be implemented in future guidelines and benefit future patients.

17 REFERENCES

1. Iqbal S, Quigley EM. Progress in Our Understanding of the Gut Microbiome: Implications for the Clinician. Curr Gastroenterol Rep. 2016;18(9):49.
2. Szajewska H, Canani RB, Guarino A, Hojsak I, Indrio F, Kolacek S, et al. Probiotics for the Prevention of Antibiotic-Associated Diarrhea in Children. J Pediatr Gastroenterol Nutr. 2016;62(3):495-506.
3. Goldenberg JZ, Lytvyn L, Steurich J, Parkin P, Mahant S, Johnston BC. Probiotics for the prevention of pediatric antibiotic-associated diarrhea. Cochrane Database Syst Rev. 2015(12):CD004827.
4. Georgieva M, Pancheva R, Rasheva N, Usheva N, Ivanova L, Koleva K. USE OF THE PROBIOTIC Lactobacillus reuteri DSM 17938 IN THE PREVENTION OF ANTIBIOTIC-ASSOCIATED INFECTIONS IN HOSPITALIZED BULGARIAN CHILDREN: A RANDOMIZED, CONTROLLED TRIAL. J Imab. 2015;21(4):895-900.
5. Jirapinyo P, Densupsoontorn N, Thamonsiri N, Wongarn R. Prevention of antibiotic-associated diarrhea in infants by probiotics. J Med Assoc Thai. 2002;85 Suppl 2:S739-42.
6. Turck D, Bernet JP, Marx J, Kempf H, Giard P, Walbaum O, et al. Incidence and risk factors of oral antibiotic-associated diarrhea in an outpatient pediatric population. J Pediatr Gastroenterol Nutr. 2003;37(1):22-6.
7. Geneva: WHOECCDIWHOTTodamfpaoshwtr.
8. Guarino A, Ashkenazi S, Gendrel D, Lo Vecchio A, Shamir R, Szajewska H, et al. European Society for Pediatric Gastroenterology, Hepatology, and Nutrition/European Society for Pediatric Infectious Diseases evidence-based guidelines for the management of acute gastroenteritis in children in Europe: update 2014. J Pediatr Gastroenterol Nutr. 2014;59(1):132-52.
9. Bartlett JG. Clinical practice. Antibiotic-associated diarrhea. N Engl J Med. 2002;346(5):334-9.
10. McFarland LV. Antibiotic-associated diarrhea: epidemiology, trends and treatment. Future Microbiol. 2008;3(5):563-78.
11. Hill C, Guarner F, Reid G, Gibson GR, Merenstein DJ, Pot B, et al. The International Scientific Association for Probiotics and Prebiotics consensus statement on the scope and appropriate use of the term probiotic. Nat Rev Gastro Hepat. 2014;11(8):506-14.
12. Ouwehand AC. A review of dose-responses of probiotics in human studies. Benef Microbes. 2017;8(2):143-51.
13. http://www.fao.org/3/a-a0512e.pdf Accessed 17 Apr 2017. FWECHanpopifipmwllab.
14. Chapman CM, Gibson GR, Rowland I. Health benefits of probiotics: are mixtures more effective than single strains? Eur J Nutr. 2011;50(1):1-17.
15. Szajewska H, Kolodziej M. Systematic review with meta-analysis: Lactobacillus rhamnosus GG in the prevention of antibiotic-associated diarrhoea in children and adults. Aliment Pharmacol Ther. 2015;42(10):1149-57.
16. Szajewska H, Kolodziej M. Systematic review with meta-analysis: Saccharomyces boulardii in the prevention of antibiotic-associated diarrhoea. Aliment Pharmacol Ther. 2015;42(7):793-801.
17. Szajewska H, Guarino A, Hojsak I, Indrio F, Kolacek S, Shamir R, et al. Use of probiotics for management of acute gastroenteritis: a position paper by the ESPGHAN Working Group for Probiotics and Prebiotics. J Pediatr Gastroenterol Nutr. 2014;58(4):531-9.
18. Koning CJ, Jonkers DM, Stobberingh EE, Mulder L, Rombouts FM, Stockbrugger RW. The effect of a multispecies probiotic on the intestinal microbiota and bowel movements in healthy volunteers taking the antibiotic amoxycillin. Am J Gastroenterol. 2008;103(1):178-89.
19. Koning CJ, Jonkers D, Smidt H, Rombouts F, Pennings HJ, Wouters E, et al. The effect of a multispecies probiotic on the composition of the faecal microbiota and bowel habits in chronic obstructive pulmonary disease patients treated with antibiotics. Br J Nutr. 2010;103(10):1452-60.

20. Lang FC. Use of a multi-species probiotic for the prevention of antibiotic associated diarrhea. Nutrafoods. 2010;9(2):27-31.
21. Hell M, Bernhofer C, Stalzer P, Kern JM, Claassen E. Probiotics in Clostridium difficile infection: reviewing the need for a multistrain probiotic. Benef Microbes. 2013;4(1):39-51.
22. Lukasik J, Szajewska H. Effect of a multispecies probiotic on reducing the incidence of antibiotic-associated diarrhoea in children: a protocol for a randomised controlled trial. BMJ Open. 2018;8(5):e021214.
23. Kotowska M, Albrecht P, Szajewska H. Saccharomyces boulardii in the prevention of antibiotic-associated diarrhoea in children: a randomized double-blind placebo-controlled trial. Aliment Pharmacol Ther. 2005;21(5):583-90.
24. Ruszczynski M, Radzikowski A, Szajewska H. Clinical trial: effectiveness of Lactobacillus rhamnosus (strains E/N, Oxy and Pen) in the prevention of antibiotic-associated diarrhoea in children. Aliment Pharmacol Ther. 2008;28(1):154-61.
25. Szajewska H, Albrecht P, Topczewska-Cabanek A. Randomized, double-blind, placebo-controlled trial: effect of lactobacillus GG supplementation on Helicobacter pylori eradication rates and side effects during treatment in children. J Pediatr Gastroenterol Nutr. 2009;48(4):431-6.
26. Hechard Y, Dherbomez M, Cenatiempo Y, Letellier F. Antagonism of lactic acid bacteria from goats' milk against pathogenic strains assessed by the ‘sandwich method’. Letters in Applied Microbiology. 1990;11(4):185-8.
27. Guarner F, Khan AG, Garisch J, Eliakim R, Gangl A, Thomson A, et al. World Gastroenterology Organisation Global Guidelines: probiotics and prebiotics October 2011. J Clin Gastroenterol. 2012;46(6):468-81.
28. Koning CJ, Jonkers DM, Stobberingh EE, Smidt H, Stockbrugger RW. Effect of a multispecies probiotic on the composition of the dominant faecal flora in healthy volunteers treated with amoxycillin. Gastroenterology. 2005;128(4):A101-A.
29. Agamennone V, Krul CAM, Rijkers G, Kort R. A practical guide for probiotics applied to the case of antibiotic-associated diarrhea in The Netherlands. BMC Gastroenterol. 2018;18(1):103.
30. Ritchie ML, Romanuk TN. A meta-analysis of probiotic efficacy for gastrointestinal diseases. PloS one. 2012;7(4):e34938-e.
31. Hempel S, Newberry S, Ruelaz A, Wang Z, Miles JN, Suttorp MJ, et al. Safety of probiotics used to reduce risk and prevent or treat disease. Evidence report/technology assessment. 2011(200):1-645.
32. Bafeta A, Koh M, Riveros C, Ravaud P. Harms Reporting in Randomized Controlled Trials of Interventions Aimed at Modifying Microbiota: A Systematic Review. Ann Intern Med. 2018;169(4):240-7.
33. Sanders ME, Akkermans LM, Haller D, Hammerman C, Heimbach J, Hormannsperger G, et al. Safety assessment of probiotics for human use. Gut Microbes. 2010;1(3):164-85.
34. Suez J, Zmora N, Zilberman-Schapira G, Mor U, Dori-Bachash M, Bashiardes S, et al. Post-Antibiotic Gut Mucosal Microbiome Reconstitution Is Impaired by Probiotics and Improved by Autologous FMT. Cell. 2018;174(6):1406-23.e16.
35. Doron S, Snydman DR. Risk and safety of probiotics. Clinical infectious diseases : an official publication of the Infectious Diseases Society of America. 2015;60 Suppl 2(Suppl 2):S129-S34.
36. Marteau P, Pochart P, Bouhnik Y, Rambaud JC. The fate and effects of transiting, nonpathogenic microorganisms in the human intestine. World Rev Nutr Diet. 1993;74:1-21.
37. Marteau P, Shanahan F. Basic aspects and pharmacology of probiotics: an overview of pharmacokinetics, mechanisms of action and side-effects. Best Pract Res Clin Gastroenterol. 2003;17(5):725-40.
38. Bezkorovainy A. Probiotics: determinants of survival and growth in the gut. Am J Clin Nutr. 2001;73(2 Suppl):399S-405S.
39. Conway PL, Gorbach SL, Goldin BR. Survival of lactic acid bacteria in the human stomach and adhesion to intestinal cells. J Dairy Sci. 1987;70(1):1-12.

40. Bouhnik Y, Pochart P, Marteau P, Arlet G, Goderel I, Rambaud JC. Fecal recovery in humans of viable Bifidobacterium sp ingested in fermented milk. Gastroenterology. 1992;102(3):875-8.
41. Blaabjerg S, Artzi DM, Aabenhus R. Probiotics for the Prevention of Antibiotic-Associated Diarrhea in Outpatients-A Systematic Review and Meta-Analysis. Antibiotics (Basel, Switzerland). 2017;6(4):21.
42. Guo Q, Goldenberg JZ, Humphrey C, El Dib R, Johnston BC. Probiotics for the prevention of pediatric antibiotic-associated diarrhea. Cochrane Database Syst Rev. 2019;4:Cd004827.
43. Lewis SJ, Heaton KW. Stool form scale as a useful guide to intestinal transit time. Scand J Gastroenterol. 1997;32(9):920-4.
44. Ghanma A, Puttemans K, Deneyer M, Benninga MA, Vandenplas Y. Amsterdam infant stool scale is more useful for assessing children who have not been toilet trained than Bristol stool scale. Acta Paediatr. 2014;103(2):e91-2.
45. Musso G, Gambino R, Cassader M. Obesity, diabetes, and gut microbiota: the hygiene hypothesis expanded? Diabetes Care. 2010;33(10):2277-84.
46. 2013;11(11):3449-n/a. doi: 10.2903/j.efsa.2013.3449 HESOotmotloQbaiatfafuEJ.
47. van den Nieuwboer M, Claassen E, Morelli L, Guarner F, Brummer RJ. Probiotic and synbiotic safety in infants under two years of age. Benef Microbes. 2014;5(1):45-60.
48. Bermudez-Brito M, Plaza-Diaz J, Munoz-Quezada S, Gomez-Llorente C, Gil A. Probiotic mechanisms of action. Ann Nutr Metab. 2012;61(2):160-74.
49. Didari T, Solki S, Mozaffari S, Nikfar S, Abdollahi M. A systematic review of the safety of probiotics. Expert Opin Drug Saf. 2014;13(2):227-39.
50. Hempel S, Newberry SJ, Maher AR, Wang Z, Miles JN, Shanman R, et al. Probiotics for the prevention and treatment of antibiotic-associated diarrhea: a systematic review and meta-analysis. JAMA. 2012;307(18):1959-69.
51. de Meij TG, Budding AE, de Groot EF, Jansen FM, Frank Kneepkens CM, Benninga MA, et al. Composition and stability of intestinal microbiota of healthy children within a Dutch population. FASEB J. 2016;30(4):1512-22.
52. Zmora N, Zilberman-Schapira G, Suez J, Mor U, Dori-Bachash M, Bashiardes S, et al. Personalized Gut Mucosal Colonization Resistance to Empiric Probiotics Is Associated with Unique Host and Microbiome Features. Cell. 2018;174(6):1388-405 e21.
53. Ricci A, Allende A, Bolton D, Chemaly M, Davies R, Gironés R, et al. Scientific Opinion on the update of the list of QPS-recommended biological agents intentionally added to food or feed as notified to EFSA2017. 177 p.
54. Severyn CJ, Bhatt AS. With Probiotics, Resistance Is Not Always Futile. Cell Host Microbe. 2018;24(3):334-6.

18 APPENDIX A: CONTACT DATA PARTICIPATING CENTRES

Amsterdam UMC, location VUmc

T.H. Dierikx
Student-researcher/PhD student, department of pediatric gastroenterology
Amsterdam UMC, location VUmc
De Boelelaan 1117, 1081 HV, Amsterdam
Email: t.dierikx@vumc.nl

dr. T.G.J. de Meij
Department of pediatric gastroenterology
Amsterdam UMC, location VUmc
De Boelelaan 1117, 1081 HV, Amsterdam
Email: t.demeij@vumc.nl

Amsterdam UMC, location AMC

dr. prof. M.A. Benninga
Department of pediatric gastroenterology
Amsterdam UMC, location AMC
Meibergdreef 9, 1105 AZ, Amsterdam
Email: m.a.benninga@amc.uva.nl

dr. T.G.J. de Meij
Department of pediatric gastroenterology
Amsterdam UMC
De Boelelaan 1117, 1081 HV, Amsterdam
Email: t.demeij@amsterdamumc.nl

Onze Lieve Vrouwe Gasthuis, locatie Oost

dr. S.R.D. van der Schoor
Department of pediatrics
Onze Lieve Vrouwe Gasthuis, locatie Oost
Oosterpark 9, 1091 AC, Amsterdam
Email: s.r.d.vanderschoor@olvg.nl

Onze Lieve Vrouwe Gasthuis, locatie West

dr. S.R.D. van der Schoor
Department of pediatrics
Onze Lieve Vrouwe Gasthuis, locatie Oost

Jan Tooropstraat 164, 1061 AE, Amsterdam
Email: s.r.d.vanderschoor@olvg.nl

19 APPENDIX B: WINCLOVE CERTIFICATE FOR SAFETY STATEMENT

NSF

NSF INTERNATIONAL

789 N. Dixboro Road, Ann Arbor, Michigan 48105 USA
+1 800 673 6275

NSF
GMP Registered
Dietary Supplements

NSF International has assessed and confirmed compliance of

Winclove Holding B.V.

Facility: Hulstweg 11, 1032 LB Amsterdam, NLD

to NSF GMP Registration Program Requirements of NSF/ANSI 173, Section 8

which includes FSMA and cGMP (21 CFR 111), (21 CFR 117)

Print Date:	December 26, 2018
Certificate Number:	C0215388-173GMPMF-1
Initial Certification:	1/29/2015 9:44:51 AM
Expiration Date:	December 31, 2019

Cheryl Luther
General Manager, Dietary Supplements and Beverage Quality

This certificate is the property of NSF International and must be returned upon request. For the most current and complete information, please access NSF's website (www.nsf.org).

Lloyd's Register

Certificate of Approval

This is to certify that the Management System of:

Winclove Holding B.V.

Hulstweg 11, 1032 LB Amsterdam, Netherlands

has been approved by LRQA to the following standards:

ISO 22000: 2005

P.G. Cornelissen
Issued By: Lloyd's Register Nederland B.V. for and on behalf of: Lloyd's Register Quality Assurance Ltd

This certificate is valid only in association with the certificate schedule bearing the same number on which the locations applicable to this approval are listed.

Current Issue Date: 22 January 2018
Expiry Date: 21 January 2021
Certificate Identity Number: 10049798

Original Approvals:
ISO 22000 – 22 January 2015

Approval Number(s): ISO 22000 – 00007517

20 APPENDIX C: SPECIFICATION OF INVESTIGATIONAL PRODUCT (Winclove 612)

Label investigational product (probiotics)

Uitsluitend bestemd voor gebruik in wetenschappelijk studieverband.
IPAD studie, Amsterdam UMC, locatie VUmc
NL69225.029.19

Ingrediënten per sachet: maïszetmeel, maltodextrines, +/- fructo-oligosacchariden (FOS) P6, +/- dextrine P9, +/- bacteriestammen (B. bifidum W23, B. lactis W51, L. acidophilus W37, L. acidophilus W55, L. paracasei W20, L. plantarum W62, L. rhamnosus W71, L. salivarius W24; ≥ 2,5*10^9 kolonievormende eenheden per gram), kaliumchloride, rijst eiwit, magnesiumsulfaat, enzymen (amylase), mangaansulfaat.

Gebruik: Neem 2x per dag de inhoud (2 gram) van 1 sachet. Los het poeder op in een glas water, borst- of flesvoeding, en laat het 1 minuut staan. Goed doorroeren voor inname. Bij voorkeur innemen op de lege maag; 1 sachet voor het ontbijt en 1 sachet voor het slapen gaan. Indien u antibiotica gebruikt: na inname van de antibiotica 2-3 uur wachten met gebruik van dit product.

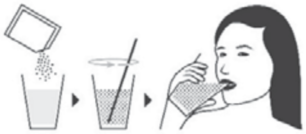

THT: zie sachet

Op kamertemperatuur in de originele verpakking bewaren.
Geschikt voor pasgeboren kinderen.
Buiten bereik van jonge kinderen houden.

Bij vragen kunt u contact opnemen met hoofdonderzoeker: dr. T.G.J. de Meij. E-mailadres: t.demeij@vumc.nl.
Telefoonnummer: +31 (0)20-4443318

Geproduceerd door Winclove Probiotics B.V., Hulstweg 11, 1032 LB Amsterdam, +31 (0)20-4350235
