# Supplementary material for: Probiotics and Antibiotic-Induced Microbial Aberrations in Children: A Secondary Analysis of a Randomized Clinical Trial
Source: JAMA Netw Open. 2024 Jul 5;7(7):e2418129. doi: 10.1001/jamanetworkopen.2024.18129 (PMC11227081; doi:10.1001/jamanetworkopen.2024.18129)
Supplement: Supplement 2. — eTable 1. Characteristics of Included Participants and Participants Lost to Follow-Up eFigure 1. Beta Diversity at All 4 Times eFigure 2. Relative Abundance at Phylum Level eFigure 3. Relative Abundance of Genera With Significantly Different Relative Abundance [file jamanetwopen-e2418129-s002.pdf]

Supplementary Online Content

Dierikx T, Malinowska A, Łukasik J, et al; Multispecies Probiotic in AAD Study Group. Probiotics and antibiotic-associated diarrhea in children: a randomized clinical trial. *JAMA Netw Open*. 2024;7(6):e2418129. doi:10.1001/jamanetworkopen.2024.18129

eTable 1. Characteristics of Included Participants and Participants Lost to Follow-Up
eFigure 1. Beta Diversity at All 4 Times
eFigure 2. Relative Abundance at Phylum Level
eFigure 3. Relative Abundance of Genera With Significantly Different Relative Abundance

This supplementary material has been provided by the authors to give readers additional information about their work.

© 2024 Dierikx T et al. *JAMA Network Open*.

eTable 1. Characteristics of Included Participants and Participants Lost to Follow-Up

	Included (n = 88)	Lost to follow up (n=225)
Age, median [IQR], months	21 [10-53]	33 [16-68]
Female sex, n (%)	34 (39)	106 (47)
Male sex, n(%)	54 (61)	119 (53)
Subjects included in the Netherlands, n (%)	72 (82)	70 (31)
Inpatient, n (%)	47 (53)	191 (84)
Hospital stay, median [IQR], days	1,5 [0-4]	5 [3-7]
Antibiotic treatment duration, median [IQR], days	7 [7-10]	10 [7-10]
Antibiotic administration route, n (%)		
Only oral	63 (72)	72 (32)
Only intravenous	5 (6)	109 (48)
Intravenous followed by oral	20 (23)	44 (20)
Reason for treatment, n (%)		
URTI	13 (15)	76 (34)
LRTI	25 (28)	69 (31)
UTI	22 (25)	34 (15)
joint	1 (1)	4 (2)
lymphadenitis	4 (5)	8 (4)
GI	2 (2)	8 (4)
Skin	13 (15)	11 (5)
NS	2 (2)	4 (2)
other	6 (7)	11 (5)
Antibiotic[b], n (%)		
2nd generation cephalosporin	5 (6)	38 (17)
3rd generation cephalosporin	7 (8)	57 (25)
Aminopenicillin	29 (33)	92 (41)
Amoxicillin with clavulanic acid	52 (50)	64 (28)
Clindamycin	1 (1)	22 (10)
other	5 (6)	13 (6)
Two concomitant antibiotics, n (%)	4 (5)	27 (12)
Change of antibiotic class, n (%)	10 (11)	35 (16)
Diarrhoea cases, n (%)[c]	20 (23)	63 (28)
AAD cases, n (%)[c]	16 (18)	35 (16)

© 2024 Dierikx T et al. *JAMA Network Open*.

eFigure 1. Beta Diversity at All 4 Times

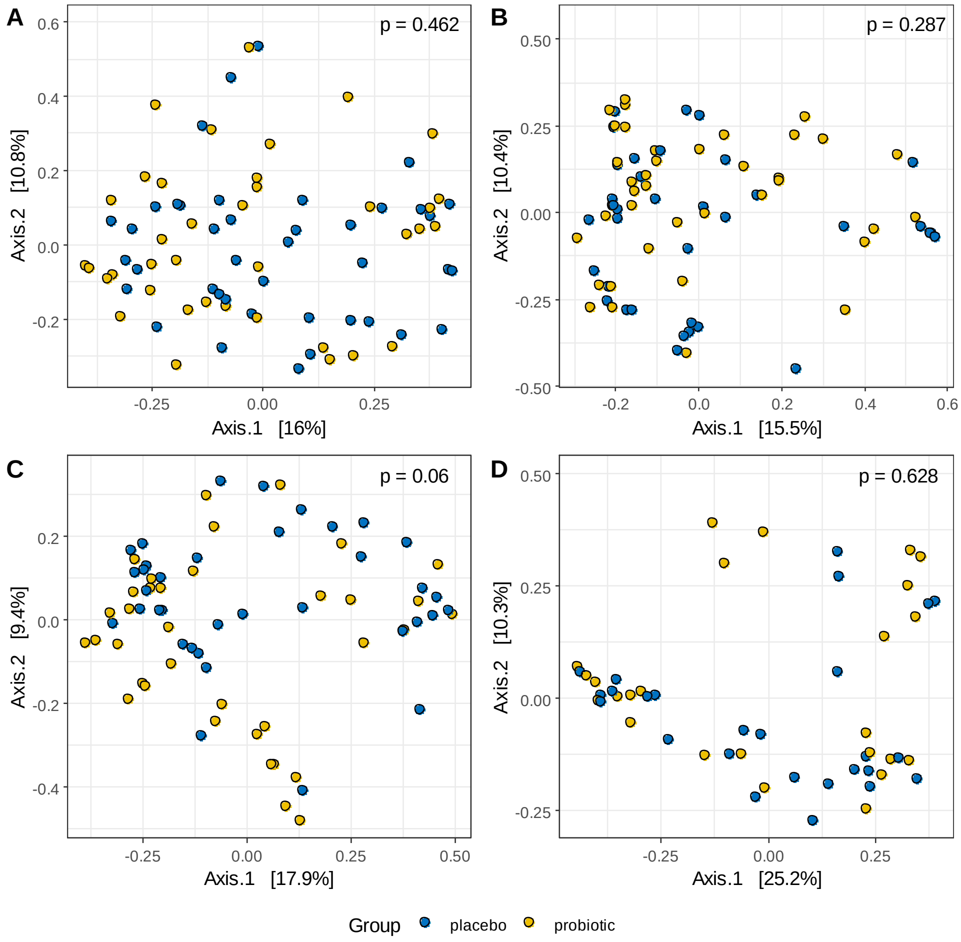

Beta-diversity is demonstrated at four time-points: At inclusion (A), at the last day of the antibiotic course (B), at the last day of the placebo or probiotic supplementation (C) and one month after the study intervention (D). There were no significant changes between the placebo group and the probiotic group at all four time-points. Axis 1 and 2 represents the first and second principal coordinate.

© 2024 Dierikx T et al. *JAMA Network Open*.

eFigure 2. Relative Abundance at Phylum Level

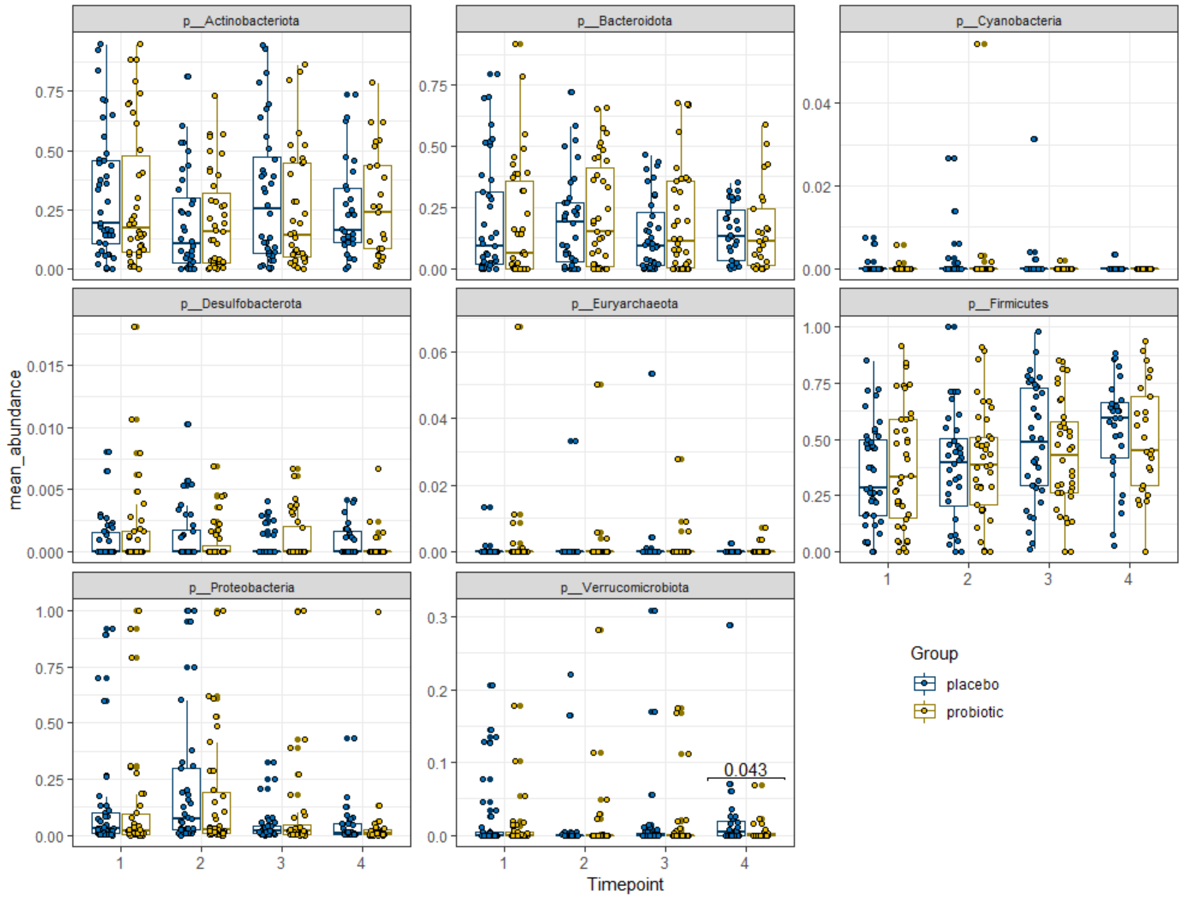

The relative abundance of the observed phyla is demonstrated at four time-points: At inclusion, at the last day of the antibiotic course, at the last day of the placebo or probiotic supplementation and one month after the study intervention. One month after the intervention, the relative abundance of Verrucomicrobiota was significantly higher in the placebo group compared to the probiotic group (p=0.043).

© 2024 Dierikx T et al. *JAMA Network Open*.

eFigure 3. Relative Abundance of Genera With Significantly Different Relative Abundance

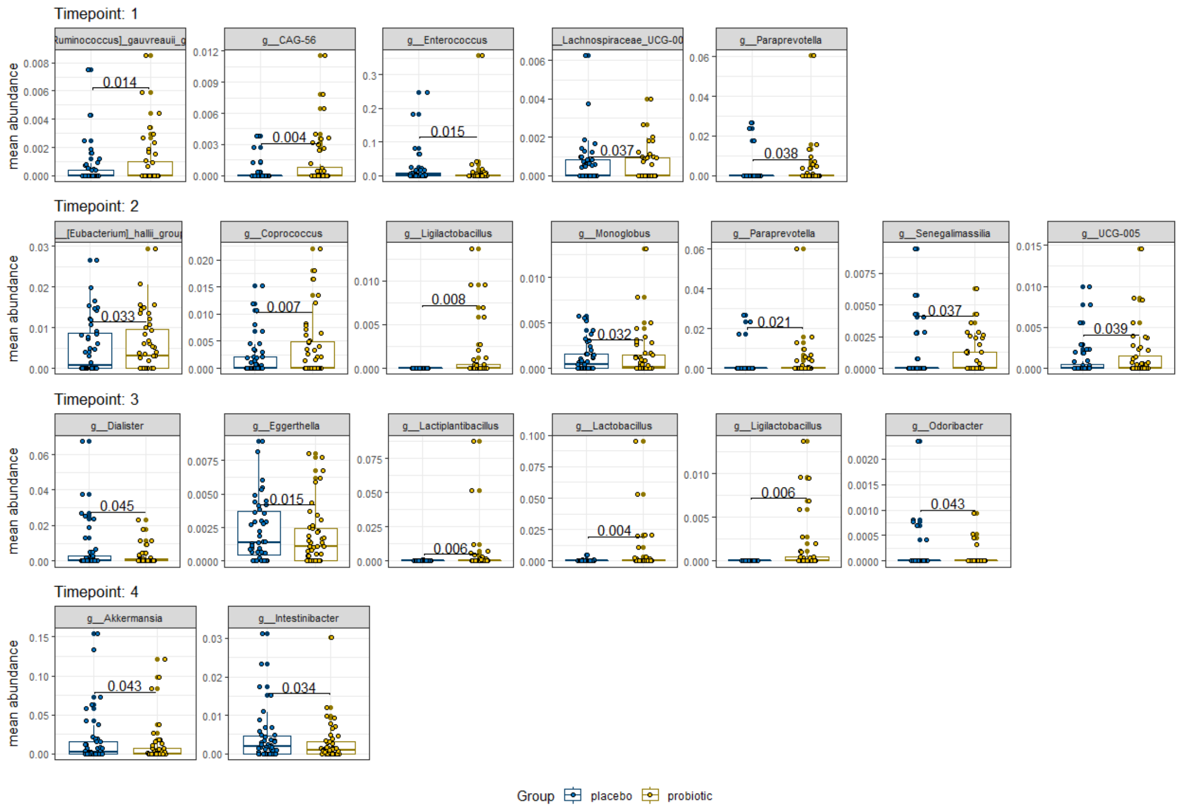

The relative abundance of the observed genera that significantly differed in relative abundance between the placebo and probiotic group is demonstrated at four time-points: At inclusion, at the last day of the antibiotic course, at the last day of the placebo or probiotic supplementation and one month after the study intervention. Significant differences were found in the abundance of 18 different taxa spread across the four time-points. An overview of all observed genera including in the placebo and probiotic group is given in supplemental dataset 1 along with adjusted p-values.

© 2024 Dierikx T et al. *JAMA Network Open*.
