# Supplementary material for: Probiotics and Antibiotic-Induced Microbial Aberrations in Children: A Secondary Analysis of a Randomized Clinical Trial
Source: JAMA Netw Open. 2024 Jul 5;7(7):e2418129. doi: 10.1001/jamanetworkopen.2024.18129 (PMC11227081; doi:10.1001/jamanetworkopen.2024.18129)
Supplement: Supplement 4. — Multispecies Probiotic in AAD Study Group [file jamanetwopen-e2418129-s004.pdf]

Supplemental Online Content: Nonauthor Collaborators
*First name, last name, and suffix (if applicable) are required and will appear in PubMed.

***Group Name(s):** Multispecies Probiotic in AAD Study Group

***First Name and Middle Initial(s)**	***Last Name**	***Suffix (eg, Jr, III)**	Academic Degrees	Institution	Location (city, state/province, country)	Role or Contribution, eg, chair, principal investigator	Group (if more than 1 Group listed in the byline) and/or Subgroup (eg, Steering Committee)
Sophie R. D.	van der Schoor		MD, PhD	OLVG Hospital	Amsterdam, Noord�Holland, The Netherlands	Helped with recruitment of participant in the OLVG hospital	
Malika	Chegary		MD, PhD	OLVG Hospital	Amsterdam, Noord�Holland, The Netherlands	Helped with recruitment of participant in the OLVG hospital	
Catharina (Karen) J.M.	Koning		PhD	Winclove Probiotics	Amsterdam, Noord�Holland, The Netherlands	Contributed in design and setup of the study, as well as on co�reading/giving input on the final manuscript.	
Mark	Hanemaaijer		PhD	Winclove Probiotics	Amsterdam, Noord�Holland, The Netherlands	Contributed in design and setup of the study, as well as on co reading/giving input on the final manuscript.	
