# Supplementary material for: Probiotics and Antibiotic-Induced Microbial Aberrations in Children: A Secondary Analysis of a Randomized Clinical Trial
Source: JAMA Netw Open. 2024 Jul 5;7(7):e2418129. doi: 10.1001/jamanetworkopen.2024.18129 (PMC11227081; doi:10.1001/jamanetworkopen.2024.18129)
Supplement: Supplement 5. — Data Sharing Statement [file jamanetwopen-e2418129-s005.pdf]

Data Sharing Statement

Dierikx. Probiotics and Antibiotic-Associated Diarrhea in Children. *JAMA Netw Open*. Published June 27, 2024. doi:10.1001/jamanetworkopen.2024.18129

Data
Data available: Yes
Data types: Deidentified participant data
How to access data: The data collected in the course of this study are available from the corresponding author upon reasonable request
When available: With publication

Supporting Documents
Document types: None

Additional Information
Who can access the data: The data collected in the course of this study are available from the corresponding author upon reasonable request
Types of analyses: Relevant to the study and working field focussing on impact of probiotics and/or antibiotics on the microbiome
Mechanisms of data availability: The data collected in the course of this study are available from the corresponding author upon reasonable request after approval of a proposal
